# Human T-Cell Leukemia Virus Type 1 (HTLV-1) Tax Requires CADM1/TSLC1 for Inactivation of the NF-κB Inhibitor A20 and Constitutive NF-κB Signaling

**DOI:** 10.1371/journal.ppat.1004721

**Published:** 2015-03-16

**Authors:** Rajeshree Pujari, Richard Hunte, Remy Thomas, Louise van der Weyden, Dan Rauch, Lee Ratner, Jennifer K. Nyborg, Juan Carlos Ramos, Yoshimi Takai, Noula Shembade

**Affiliations:** 1 Department of Microbiology and Immunology, Viral Oncology Program, Sylvester Comprehensive Cancer Center, Miller School of Medicine, The University of Miami, Miami, Florida, United States of America; 2 Wellcome Trust Sanger Institute, Wellcome Trust Genome Campus, Hinxton, Cambridge, United Kingdom; 3 Department of Medicine, Division of Molecular Oncology, Washington University School of Medicine, Saint Louis, Missouri, United States of America; 4 Department of Biochemistry and Molecular Biology, Colorado State University, Fort Collins, Colorado, United States of America; 5 Department of Medicine, Division of Hematology-Oncology, Viral Oncology Program, Sylvester Comprehensive Cancer Center, and Center for AIDS Research and Department of Microbiology and Immunology, University of Miami Miller School of Medicine, Miami, Florida, United States of America; 6 Department of Biochemistry and Molecular Biology, Kobe University Graduate School of Medicine, Chuo-ku, Kobe, Japan; University of Pennsylvania School of Medicine, UNITED STATES

## Abstract

Persistent activation of NF-κB by the Human T-cell leukemia virus type 1 (HTLV-1) oncoprotein, Tax, is vital for the development and pathogenesis of adult T-cell leukemia (ATL) and HTLV-1-associated myelopathy/tropical spastic paraparesis (HAM/TSP). K63-linked polyubiquitinated Tax activates the IKK complex in the plasma membrane-associated lipid raft microdomain. Tax also interacts with TAX1BP1 to inactivate the NF-κB negative regulatory ubiquitin-editing A20 enzyme complex. However, the molecular mechanisms of Tax-mediated IKK activation and A20 protein complex inactivation are poorly understood. Here, we demonstrated that membrane associated CADM1 (Cell adhesion molecule1) recruits Ubc13 to Tax, causing K63-linked polyubiquitination of Tax, and IKK complex activation in the membrane lipid raft. The c-terminal cytoplasmic tail containing PDZ binding motif of CADM1 is critical for Tax to maintain persistent NF-κB activation. Finally, Tax failed to inactivate the NF-κB negative regulator ubiquitin-editing enzyme A20 complex, and activate the IKK complex in the lipid raft in absence of CADM1. Our results thus indicate that CADM1 functions as a critical scaffold molecule for Tax and Ubc13 to form a cellular complex with NEMO, TAX1BP1 and NRP, to activate the IKK complex in the plasma membrane-associated lipid rafts, to inactivate NF-κB negative regulators, and maintain persistent NF-κB activation in HTLV-1 infected cells.

## Author Summary

HTLV-1 infection leads to the development of Adult T-cell Leukemia (ATL) or HTLV-1 associated myelopathy/ tropical spastic paraparesis (HAM/TSP). One of the major causes responsible for the development of HTLV-1 associated diseases is chronic inflammation directed by NF-kappaB (NF-κB). NF-κB activation in response to a wide variety of signals is transient and tightly controlled by ubiquitin-editing enzyme A20. One of the mechanisms of persistent NF-κB activation in HTLV-1 infected cells is inactivation of NF-κB negative regulators; however, the precise mechanism is unknown. Here, we focused on host tumor suppressor Cell adhesion molecule 1 (CADM1) that is robustly upregulated in HTLV-1 infected cells. The expression of CADM1 is frequently silenced in several cancers; however, it is critical for HTLV-1 associated ATL tumor cell survival. We characterized the role of CADM1 in persistent NF-κB activation in HTLV-1 infected cells. We found that CADM1 is required for the HTLV-1 oncoprotein, Tax, to form a cellular complex with Ubc13, TAX1BP1, NRP and NEMO in the membrane lipid rafts micorodomain. We further demonstrated that Tax requires CADM1 to inactivate NF-κB negative regulator and maintain persistent NF-κB activation. Our study reveals a novel mechanism of chronic NF-κB activation by CADM1 in HTLV-1 infected cells.

## Introduction

Infection with human T-cell leukemia virus type 1 (HTLV-1), an oncogenic retrovirus, is associated with the development of adult T-cell leukemia (ATL), an aggressive and lethal malignancy of CD4^+^ T lymphocytes and a chronic neuroinflammatory disease termed HTLV-1-associated myelopathy/tropical spastic paraparesis (HAM/TSP). HTLV-1 encodes a 40-kDa oncoprotein Tax that regulates viral gene expression and plays vital roles in ATL leukemogenesis [1–3]. Tax regulates the expression of viral and cellular genes involved in cell transformation, immortalization, and tumor initiation through NF-κB, cyclic AMP response element-binding protein (CREB), and serum responsive factor (SRF) signaling pathways [4,5]. Tax also promotes cellular transformation by inducing post-translational modifications of multiple cellular factors, inactivating tumor suppressors, and dysregulating cellular signaling pathways and cell cycle machinery [6–12]. The carboxyl-terminal PDZ-binding domain motif (PBM) of Tax recruits PDZ domain-containing cellular factors, which play critical roles in the dysregulation of signaling pathways, proliferation, and immortalization of primary T-cells [13]. One of the key functions of Tax is the persistent activation of the nuclear factor kappa-B (NF-κB) transcription factor signaling pathways that are important for transformation, proliferation, and survival of HTLV-1 infected T-cells [14–16]. Tax also maintains persistent NF-κB activation by inactivating NF-κB negative regulators, such as A20 and cylindromatosis (CYLD) [17–19]. However, the underlying mechanisms of Tax-mediated inactivation of NF-κB negative regulators and persistent NF-κB activation remain poorly understood.

NF-κB plays critical roles in inflammation and the development of innate and adaptive immunity [20]. The NF-κB family is composed of five members, NF-κB1 (p50/p105), NF-κB2 (p52/p100), p65 (RelA), RelB, and c-Rel, and each of these proteins can form homo- and heterodimers [21]. Upon stimulation of TNF receptor 1 (TNFR1) with TNF or the T-cell receptor (TCR) with antigen, NF-κB activation is triggered in the membrane microdomains, termed lipid rafts [22,23]. NF-κB is sequestered in the cytoplasm as an inactive form by the family of IκB proteins and can be rapidly activated in response to stimulation [24]. NF-κB activating signals converge at the IκB kinase (IKK) complex containing the catalytic kinase subunits IKKα, IKKβ, and the regulatory subunit IKKγ (also known as NEMO) [25]. Based on the involvement of specific receptors and extracellular stimuli, NF-κB pathways are classified into either canonical (classical) or noncanonical (alternative) pathways. The classical NF-κB pathway is dependent on IKKβ and NEMO, whereas the alternative NF-κB pathway is dependent on IKKα.

In the classical NF-κB pathway, IKKβ phosphorylates IκBα on two serine residues in a NEMO-dependent manner which triggers its polyubiquitination followed by proteasome degradation of IB, thus liberating NF-κB and allowing its rapid mobilization into the nucleus where it regulates the expression of a plethora of genes involved in cell growth, inflammation and survival [26]. In the alternative NF-κB pathway, IKKα is activated by the NF-κB inducing kinase (NIK) in response to specific ligands of the TNF superfamily, including BAFF, lymphotoxin- and CD40L [27]. Activated IKKα phosphorylates p100 that results in the ubiquitination and partial degradation of p100 by the proteasome to generate p52. Tax constitutively activates both the classical and alternative NF-κB pathways by interacting with NEMO in the absence of extracellular stimuli [28]. It has been demonstrated that Ubc13-dependent Tax ubiquitination is essential for interaction with NEMO [15]. Interestingly, Tax-mediated NF-κB activation is initiated in the Golgi-associated lipid rafts [29]; however, the mechanism is poorly understood. It has also been recently demonstrated that Tax requires Tax1 binding protein1 (TAX_1_BP_1_) to activate NF-κB [19,30]. TAX_1_BP_1_ is an adaptor molecule for the NF-κB negative regulatory ubiquitin-editing enzyme A20 complex in receptor mediated NF-κB signaling pathways [31,32]. Phosphorylation of TAX_1_BP_1_ by IKKα in a stimulus-dependent manner assembles the ubiquitin-editing enzyme A20 complex (A20, TAX1BP1, Itch and RNF11) to terminate NF-B signaling and thus maintain transient NF-κB activation. Interestingly, TAX_1_BP_1_-mediated assembly of the A20 ubiquitin-editing enzyme complex was impaired due to the disruption of TAX_1_BP_1_ and IKKα interactions by Tax [19] in TNF- or IL-1-stimulated cells. However, the mechanism of TAX_1_BP_1_ and IKKα dissociation in Tax expressing cells is poorly understood.

Cell adhesion molecule 1 (CADM1; also known as TSLC1, IGSF4 or NECL2) is encoded by chromosomal region 11q23 and was first identified as a tumor suppressor gene in non-small cell lung cancer (NSCLC) [33]. CADM1 is a 442 amino acid protein that is highly upregulated in ATL cells [34]. Although CADM1 is localized in the cytoplasm and cell membrane, it is mostly localized in the basolateral membrane of the cells [35–39]. The N-terminal three extracellular Ig-like loops and the C-terminal cytoplasmic tail are the main functional regions of the CADM1 protein. The extracellular loops interact with the EGF receptor/ErbB family members, cell surface receptor class-I-restricted T-cell-associated molecule (CRTAM), and integrin α6β4 [40–42] and the cytoplasmic tail contains a protein 4.1-binding motif (protein 4.1-BM) and a type II PDZ-binding motif (PDZ-BM) which are critical for CADM1 function [43]. CADM1 plays a critical role in the adhesion of spermatogenic cells to Sertoli cells [44]. The interaction between CRTAM expressed on activated cytotoxic T-cells and CADM1 expressed on antigen presenting cells drives the production of IFN-γ and IL-22 by the activated CD8^+^ T-cells [45]. Studies with CADM1-deficient mice have revealed that the interaction between CADM1 and the TCR ζ-chain is critical for T-cell functions [46]. It was also demonstrated that CADM1 expression is reduced in lung cancer cell lines and the reintroduction of CADM1 into A549, a NSCLC cell line, significantly inhibited tumorigenicity in nude mice [47]. Intriguingly, although CADM1 functions as a tumor suppressor in NSCLC, it may function as an oncoprotein in ATL cells [47]. The fundamental mechanistic role of CADM1 as an oncoprotein in ATL cell is not known.

Here we demonstrated that the expression of CADM1 was regulated by Tax. CADM1 was required for K63-linked polyubiqutination of Tax in the membrane lipid rafts. Interestingly, CADM1 was also required for Tax to interact with Ubc13, NEMO, TAX_1_BP_1_, and NRP, and for activation of the IKK complex in the membrane-associated lipid raft in HTLV-1 infected T-cells. Finally, Tax failed to inhibit TAX_1_BP_1_ phosphorylation, which is critical for the assembly of the NF-κB negative regulator, ubiquitin-editing enzyme A20 complex, in *Cadm1*-deficient MEFs stimulated with TNF or IL-1, indicating that CADM1 is not only required for Tax to activate NF-κB but also to target NF-κB negative regulators in HTLV-1 mediated tumor cells.

## Results

### The expression of CADM1 is upregulated in Tax-expressing cells

**Fig 1:**
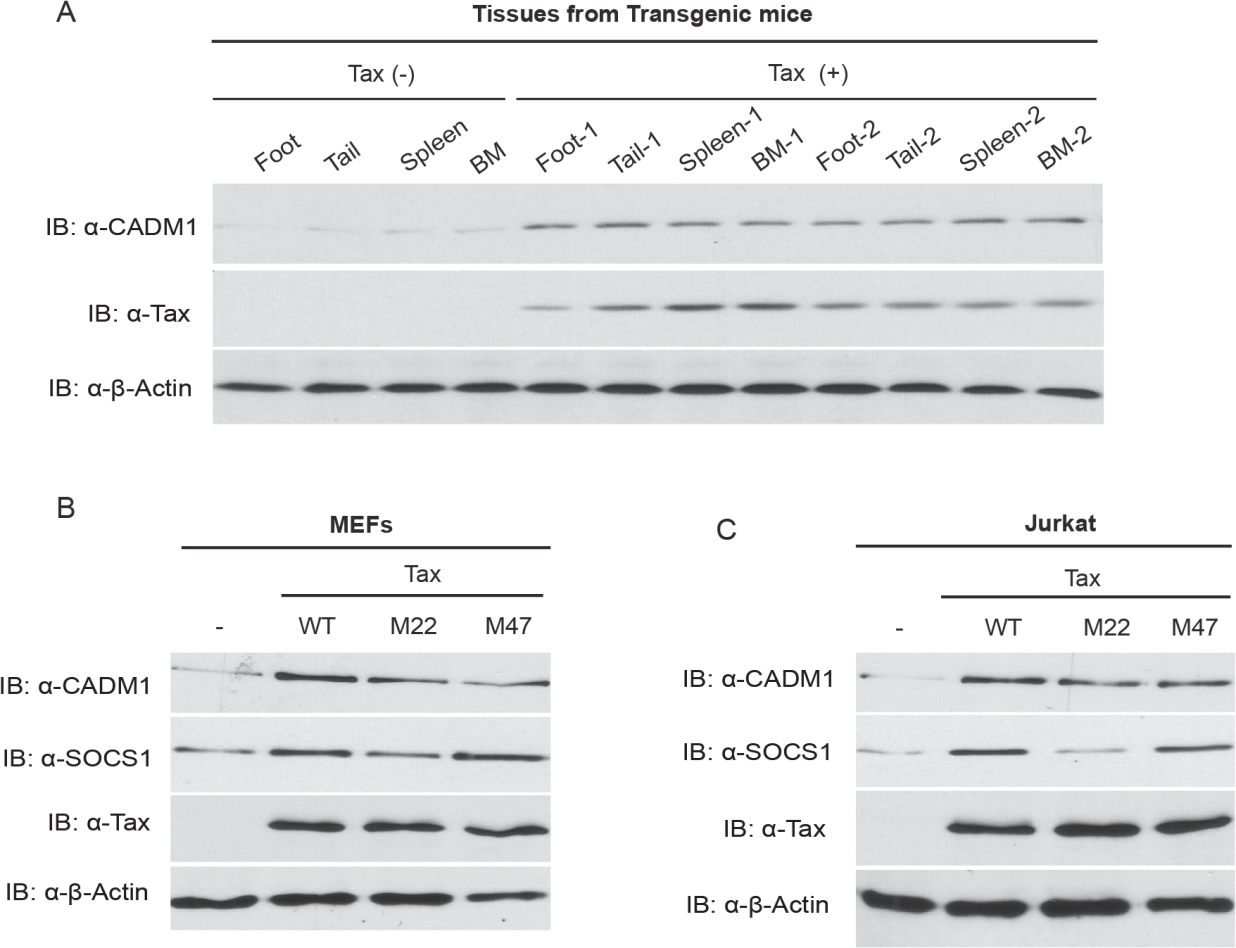
Tax induces CADM1 expression. (A) CADM1 expression in foot, tail, spleen and bone marrow (BM) tissues derived from spontaneous tumors of 14 month old control Tax-negative and Tax-positive transgenic mice. Immunoblotting was performed with anti-CADM1, Tax, and β-actin antibodies. CADM1 expression in lentiviral-transduced empty vector wildtype Tax or Tax mutants (M22 or M47) in primary MEFs (B) and Jurkat T-cells (C) was analyzed with anti-CADM1, SOCS1, Tax, and β-actin antibodies.

The expression of CADM1 has been shown to be upregulated in HTLV-1 transformed and primary ATL cells derived from acute type ATL patients; however, the mechanism of CADM1 regulation in HTLV-1 infected cells is currently unknown [34,48]. The Tax point mutant M47 activates NF-κB (but not CREB/ATF), and Tax M22 activates ATF/CREB (but not NF-κB) transcription factors [10]. Recent findings have showed massive inflammation and spontaneous tumor development at different sites in Tax transgenic mice [49,50]. Therefore, we first examined CADM1 expression in the spontaneous tumors that developed in Tax transgenic mice, and found that all tumors from these mice displayed elevated levels of CADM1 expression compared to normal mice (Tax-negative control mice) (Fig. 1A). Lentiviral-mediated expression of wildtype Tax, and Tax mutants M22 and M47 showed increased CADM1 mRNA (S1A-S1B Fig.) and protein expression in primary murine embryonic fibroblasts (MEFs) and Jurkat T-cells (Fig. 1B-C). Interestingly, lentiviral expressing Tax double mutant M22 and M47, that could activate neither CREB nor NF-κB, failed to induce CADM1 protein expression in MEFs cells (S7 Fig.). Recent reports suggest that the expression of suppressor of cytokine signaling 1 (SOCS1) is regulated by Tax in HTLV-1 infected cells through NF-κB but not through the CREB pathway [51,52], and we found that wildtype Tax and the Tax mutant M47, but not the Tax mutant M22, induce the expression of SOCS1 mRNA (S1A-S1B Fig.) and protein (Fig. 1B-C). We also observed elevated CADM1 protein expression in Tax expressing HTLV-1 infected C8166, MT-2 and MT-4 cells as compared to Jurkat T-cells (S1C Fig.). Collectively, these results suggest that Tax regulates the expression of CADM1 through the mechanisms dependent of NF-κB and CREB pathways.

### Tax interacts with the cytoplasmic tail of CADM1

**Fig 2:**
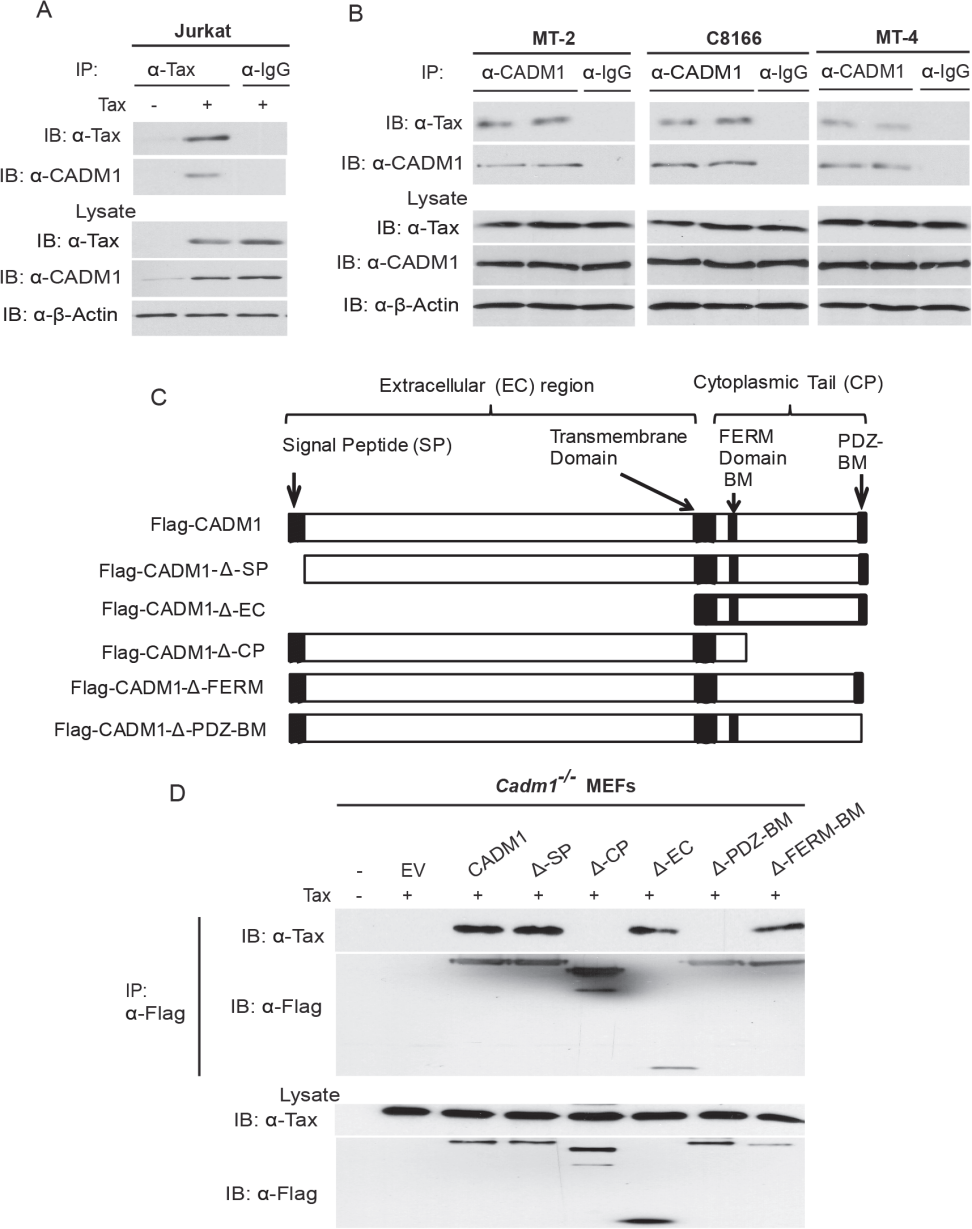
Tax interacts with CADM1. (A) Jurkat T-cells were transduced with Tax-expressing lentiviruses. After 48 hours, cells were lysed and immunoprecipitated with either anti-Tax or control anti-IgG, followed by immunoblotting with anti-Tax and anti-CADM1 antibodies. Lysates were examined for Tax, CADM1, and β-actin expression. (B) Lysates from Tax expressing HTLV-1 transformed C8166, MT-2 and MT-4 cells were immunoprecipitated in duplicate with either anti-CADM1 or control anti-IgG, followed by immunoblotting with anti-Tax and anti-CADM1. Lysates were examined for Tax, CADM1, and β-actin expression. (C) A schematic overview of the FLAG-CADM1 deletion mutants ΔSP, ΔCP, ΔEC, ΔPDZ-BM and ΔFERM. (D) Mapping the interaction between CADM1 and Tax. Primary *Cadm1* ^*−/−*^ MEFs were transfected with Tax expression vector with the indicated Flag-CADM1 mutants. After Thirty-six hours post-transfection, proteins from lysates were immunoprecipitated with anti-Flag and detected by immunoblotting with anti-Tax and anti-Flag antibodies. Lysates were immunoblotted with anti-Tax, and anti-Flag antibodies.

To gain more insight into the regulation of CADM1 expression by Tax, we performed co-immunoprecipitation (Co-IP) assays to determine if there was a physiologically relevant association of Tax with CADM1 in Jurkat T-cells. Tax was expressed by lentiviral transduction and was subsequently immunoprecipitated with anti-Tax using JurkaT-cell lysate. Surprisingly, Tax interacted with endogenous CADM1 (Fig. 2A); however, no binding was observed when immunoprecipitations were performed with a control mouse immunoglobulin antibody (Fig. 2A). We also detected the interaction between Tax and CADM1 in HTLV-1 transformed cell lines C8166, MT-2 and MT-4 proving that Tax indeed physically associated with CADM1 in HTLV-1 infected T-cells (Fig. 2B). These results suggest that Tax is tightly and stably associated with CADM1. We generated several CADM1 deletion mutants to examine the Tax interacting region (Fig. 2C). *Cadm1* ^*−/−*^ MEFs were reconstituted with these Flag-tagged CADM1 deletion mutants to determine the domain(s) important for Tax interaction. Deletion of the extracellular region (Δ-EC) had no effect on the Tax and CADM1 interaction (Fig. 2D). However, deletion of the cytoplasmic tail (Δ-CP), specifically PDZ-binding motif (Δ-PDZ-BM) abrogated CADM1 and Tax interaction (Fig. 2D). These studies revealed that the PDZ binding motif of the cytoplasmic tail of CADM1, but not the extracellular region, is critical for Tax interaction.

### CADM1 is required for Tax to interact with its adaptor molecules

Since previous studies showed that Tax interaction with its adaptor molecules, TAX_1_BP_1,_ NEMO, NEMO-Related Protein (NRP), and the ubiquitin-conjugating enzyme Ubc13, is essential for the activation of NF-κB [15,19,53], we next examined whether CADM1 was required for Tax to interact with these adaptor molecules. To address this possibility, HTLV-1 transformed MT-2 cells were stably transduced with four distinct CADM1 shRNAs and CADM1 knockdown was confirmed by immunoblotting, which revealed that shRNA number 3 was the most efficient in the knockdown of endogenous CADM1 expression in MT-2 cells (S2A Fig.). We next examined by co-IPs the endogenous interactions of Tax with TAX_1_BP_1,_ NEMO, NRP and Ubc13 in the HTLV-1 transformed C8166, MT-2 and MT-4 cell lines expressing control or CADM1 shRNA. Tax interaction with endogenous TAX_1_BP_1,_ NEMO, NRP and Ubc13 occurred in the presence of control shRNA, but not CADM1 shRNA (S2B Fig.). Similarly, Tax interacted with its adaptor molecules in *Cadm1* ^*+/+*^ but not in *Cadm1* ^*−/−*^ MEFs (S2C Fig.). Collectively, these results suggest that CADM1 is required for Tax to interact with its adaptor molecules TAX_1_BP_1,_ NEMO, NRP and Ubc13.

### CADM1 is required for Tax K63-linked polyubiquitination

**Fig 3:**
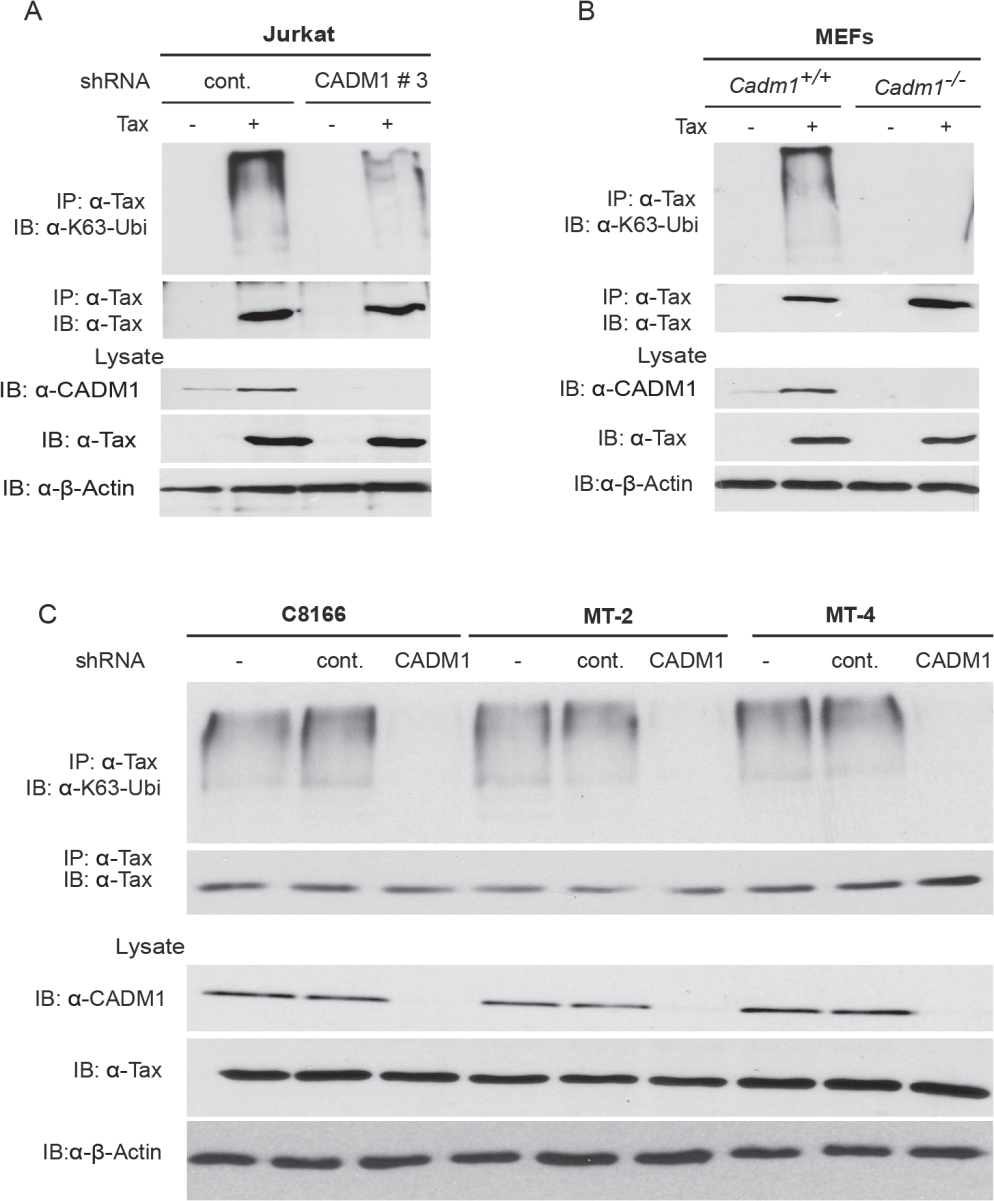
CADM1 is required for Tax K63-linked polyubiquitination. (A) Lentiviral Tax was transduced in Jurkat T-cells stably expressing control scrambled shRNA or CADM1 shRNA. After 48 hours, cells were lysed and immunoprecipitated with anti-Tax, followed by immunoblotting with anti-Ubi-K63 and anti-Tax antibodies. Lysates were examined for Tax, CADM1 and β-actin expression. (B) Lentiviral Tax was transduced in *Cadm1* ^*+/+*^ and *Cadm1* ^*−/−*^ MEFs. After 48 hours cells were lysed and immunoprecipitated with anti-Tax followed by immunoblotting with anti-K63-ubi and anti-Tax antibodies. Lysates were examined for Tax, CADM1, and β-actin expression. (C) Lysates from HTLV-1 transformed (C8166, MT-2, and MT-4) cells stably expressing CADM1 shRNA were immunoprecipitated with anti-Tax followed by immunoblotting with anti-K63-ubi and anti-Tax antibodies. Lysates were examined for Tax, CADM1, and β-actin expression.

The mechanistic and functional role of the cytoplasmic tail of CADM1 has not been well understood. Several reports have documented that K63-linked polyubiquitination of Tax is indispensable for its activity and interaction with NEMO and NRP, and subsequent activation of the NEMO/IKK complex [15,53]. Therefore, we next analyzed Tax K63-linked polyubiquitination in Jurkat T-cells transduced with lentiviruses expressing Tax and either control or CADM1 shRNA number 3 (hereafter referred to as CADM1 shRNA). Lysates were immunoprecipitated with Tax antibody, eluted with 1% SDS, diluted in lysis buffer and re-immunoprecipitated with Tax antibody to ensure that we were examining Tax ubiquitination, and not that of an associated protein. Tax polyubiquitination was detected by immunoblotting with antibodies to K63-Ubi or Tax. Knockdown of CADM1 greatly reduced the K63-linked polyubiqutination of Tax compared to cells expressing control shRNA (Fig. 3A). CADM1 knockdown had no effect on the expression of Tax as confirmed by immunoblotting (Fig. 3A). We next examined the K63-linked polyubiquitination of Tax in *Cadm1* ^*−/−*^ MEFs. The Tax K63-linked polyubiquitination assay was carried out as above using lysates from *Cadm1* ^*+/+*^ and *Cadm1* ^*−/−*^ MEFs transduced with Tax-expressing lentiviruses. The K63-linked polyubiquitination of Tax was markedly impaired in *Cadm1* ^*−/−*^ MEFs compared to *Cadm1* ^*+/+*^ control cells (Fig. 3B). Furthermore, knockdown of endogenous CADM1 with lentiviral shRNA in C8166, MT-2 and MT-4 cells also showed loss of K63-linked polyubiquitination of endogenous Tax (Fig. 3C). These collective results strongly suggest that CADM1 is absolutely essential for K63-linked polyubiquitination of Tax. Since CADM1 does not possess ubiquitin-conjugating or ubiquitin-ligase enzyme activity, this suggests that it may be recruiting E2 ubiquitin-conjugating enzyme Ubc13 on Tax.

### CADM1 is required for Tax activation of NF-κB

**Fig 4:**
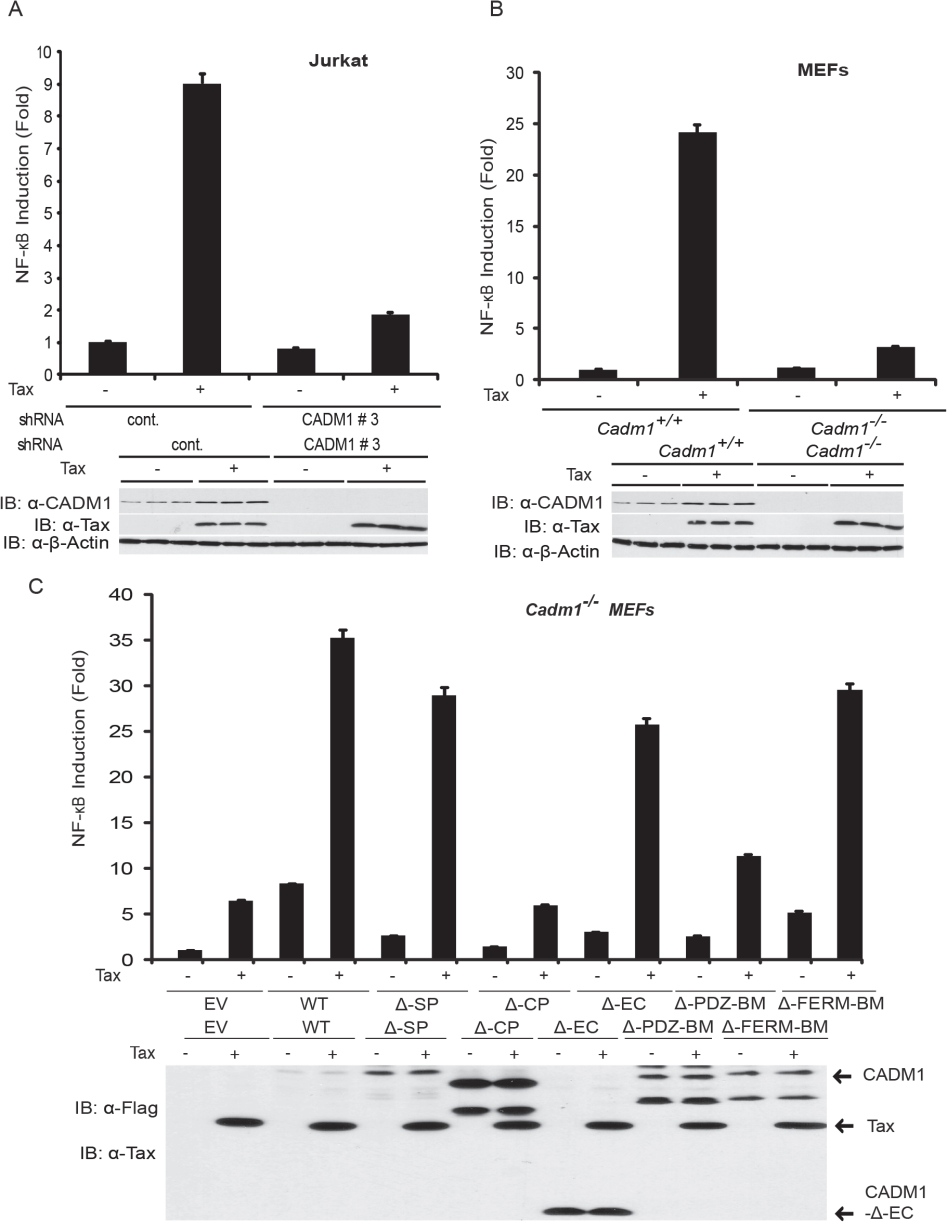
Tax-mediated NF-κB activation is impaired in *Cadm1* ^*−/−*^ MEFs. (A) NF-κB luciferase assay using lysates of Jurkat T-cells stably expressing control scrambled shRNA or CADM1 shRNA and transfected with pRL-tk internal control *Renilla* luciferase plasmid, κB-TATA Luc and Tax as indicated. After 48 hours, lysates were subjected to dual luciferase assays. The lysates were also subjected to immunoblotting to examine Tax, CADM1, and β-actin expression. (B) NF-κB luciferase assay using lysates of *Cadm1* ^*+/+*^ and *Cadm1* ^*−/−*^ MEFs transfected with pRL-tk internal control *Renilla* luciferase plasmid, κB-TATA Luc and Tax as indicated. The lysates were also subjected to immunoblotting to examine the Tax, CADM1, and β-actin expression. (C) NF-κB luciferase assay using lysates of *Cadm1* ^*−/−*^ MEF*s* transfected with a pRL-tk internal control *Renilla* luciferase plasmid, κB-TATA Luc plus empty vector or an expression vector for Flag-tagged wildtype CADM1, ΔSP, ΔCP, ΔEC, ΔPDZ-BM and ΔFERM with (+) or without (−) a plasmid encoding Tax. The lysates were also subjected to immunoblotting to examine expression of Tax and Flag for wildtype deletion mutants of CADM1. Error bars represent s.e.m. of triplicates.

**Fig 5:**
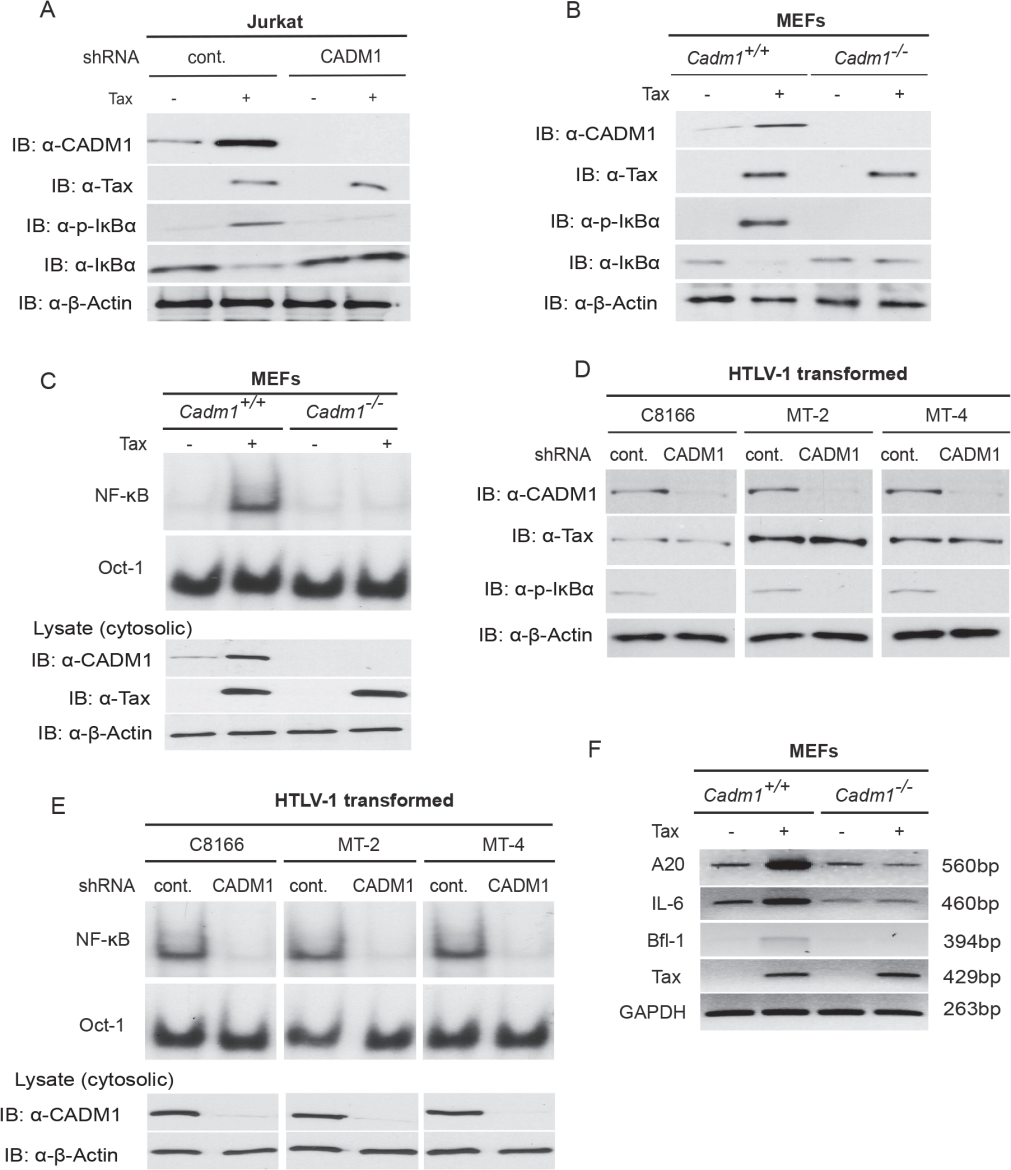
Tax requires CADM1 for NF-κB activation. (A) Lentiviral Tax was transduced in Jurkat T-cells stably expressing control scrambled shRNA or CADM1 shRNA. After 48 h, lysates were subjected to immunoblotting with anti-IκBα, anti-phospho-IκBα, anti-CADM1, anti-Tax, and anti-β-actin antibodies. (B) Primary *Cadm1* ^*+/+*^ and *Cadm1* ^*−/−*^ MEFs were transduced with Tax-expressing lentiviruses. After 48 h, lysates were subjected to immunoblotting with anti-IκBα, anti-phospho-IκBα, anti-CADM1, anti-Tax, and anti-β-actin antibodies. (C) Nuclear extracts from lentiviral expressing Tax in primary *Cadm1* ^*+/+*^ and *Cadm1* ^*−/−*^ MEFs were used for NF-κB and Oct-1 EMSA, and cytoplasmic extract were subjected to immunoblotting with anti-Tax, anti-CADM1, and anti-β-actin antibodies. (D) Lysates from HTLV-1 transformed C8166, MT-2, and MT-4 cells stably expressing control scrambled shRNA or CADM1 shRNA were subjected to immunoblotting with anti-IκBα, anti-phospho-IκBα, anti-CADM1, anti-Tax, and anti-β-actin antibodies. (E) Nuclear extracts from HTLV-1 transformed C8166, MT-2, and MT-4 cells stably expressing control scrambled shRNA or CADM1 shRNA were used for NF-κB and Oct-1 EMSA, and cytoplasmic extracts were subjected to immunoblotting with anti-CADM1 and anti-β-actin antibodies. (F) Primary *Cadm1* ^*+/+*^ and *Cadm1* ^*−/−*^ MEFs were transduced with Tax-expressing lentiviruses as described for panel B. After 48 hours, RNA was prepared and subjected to RT-PCR for A20, IL-6, Bfl-1, Tax, and GAPDH expression.

Previous studies have demonstrated that K63-linked polyubiquitination of Tax is critical for activation of NF-κB pathways, and Tax also activates noncanonical NF-κB pathways [15,53]. We therefore examined the functional role of CADM1 in both the canonical (NF-κB1) and noncanonical (NF-κB2) NF-κB activation pathways by Tax. First, to examine canonical NF-κB activation, endogenous CADM1 expression was stably suppressed with lentiviral shRNA in Jurkat T-cells, which were then transfected with Tax and NF-κB luciferase plasmids. NF-κB activation was examined by both luciferase assays and immunoblotting for phosphorylated IκBα. As expected, Tax-mediated activation of NF-κB was impaired in CADM1 knockdown Jurkat T-cells as determined by luciferase assays (Fig. 4A) and phosphorylation of IκBα (Fig. 5A). Similarly, induction of IκBα phosphorylation, and NF-κB DNA binding were impaired in *Cadm1* ^*−/−*^ MEFs compared to wildtype MEFs (Figs. 4B, 5B). A control Oct-1 EMSA demonstrated similar DNA binding in all of the nuclear extracts (Fig. 5C). We next examined the motifs of CADM1 that were required for Tax to activate canonical NF-κB. Using primary *Cadm1* ^*−/−*^ MEFs transfected with Tax, in the absence or presence of wildtype or deletion mutants of CADM1, we found that the overexpression of CADM1 alone induced NF-κB more robustly than Tax and the cytoplasmic tail and PDZ-BM of CADM1 were required for Tax to activate canonical NF-κB (Fig. 4C). Phosphorylation of IκBα and NF-κB DNA binding were also abrogated in HTLV-1 transformed C8166, MT-2 and MT-4 cells (Fig. 5D, E) after knockdown of CADM1 by CADM1 shRNA, suggesting that constitutive NF-κB activation in these cells was CADM1-dependent. Finally, the induction of NF-κB target genes by Tax was examined in *Cadm1* ^*+/+*^ and *Cadm1* ^*−/−*^ MEFs by RT-PCR. Induction of NF-κB target genes, A20, IL-6, and Bfl-1 by Tax were defective in *Cadm1* ^*−/−*^ MEFs (Fig. 5F). These results suggest that CADM1 is required for Tax to activate the canonical NF-κB pathway.

Next, we examined if CADM1 was required for Tax to activate the noncanonical NF-κB pathway. When Tax was overexpressed in Jurkat T-cells that were stably knocked down for CADM1, we found that Tax-mediated processing of p100 to p52 was completely impaired in CADM1 knockdown cells compared to cells expressing control shRNA (S3A Fig.). We also confirmed that Tax-mediated processing of p100 to p52 was impaired in *Cadm1*-deficient MEFs (S3B Fig.). Thus, CADM1 is essential for Tax to activate both the canonical and noncanonical NF-κB pathways.

### CADM1 is required for Tax to activate IKK in the plasma membrane lipid rafts

**Fig 6:**
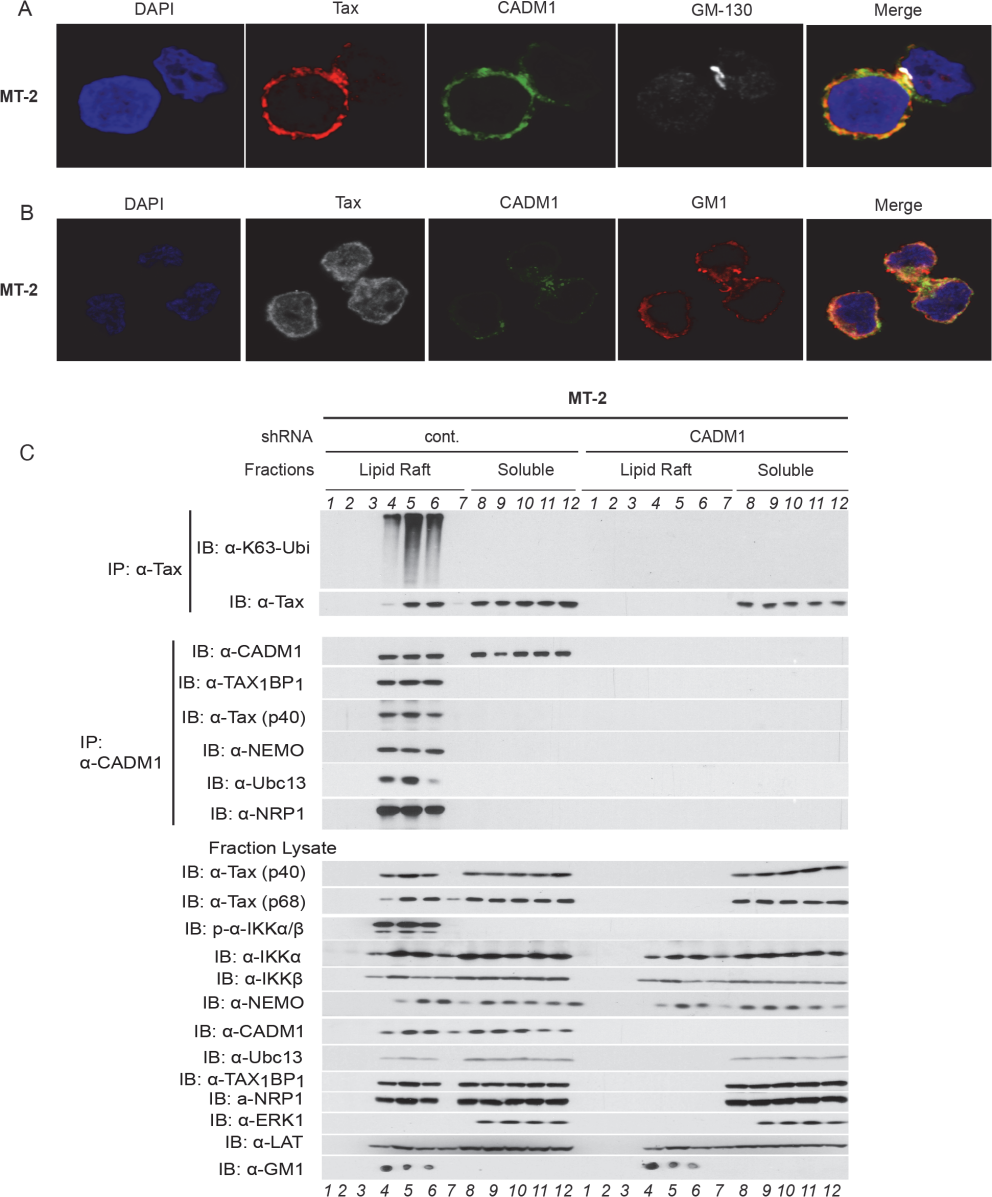
Membrane associated CADM1 mediates K63-linked polyubiquitination of Tax and links Tax adaptor proteins in the lipid rafts. (A) MT-2 cells were stained with DAPI, anti-Tax, anti-CADM1, and anti-GM-130, and subjected to confocal microscopy. (B) MT-2 cells were stained with DAPI, anti-Tax, anti-CADM1, and cholera toxin B conjugated with red fluorescence to detect GM-1, and subjected to confocal microscopy. (C) Lipid raft fractionations from MT-2 cells stably expressing control scrambled shRNA or CADM1 shRNA were split into half and subjected to immunoprecipitation with either anti-Tax or anti-CADM1. Samples immunoprecipitated with anti-Tax were immunoblotted with anti-K63-ubi and anti-Tax. Samples immunoprecipitated with anti-CADM1 were immunoblotted with anti-CADM1, anti-TAX_1_BP_1_, anti-Tax, anti-NEMO, anti-Ubc13, and anti-NRP antibodies. Lysates from lipid rafts fractions were examined for Tax, phospho-IKKα/β, total IKKα, IKKβ, NEMO, CADM1, Ubc13, TAX_1_BP_1_, NRP, ERK1 (marker for soluble fractions), LAT (lipid raft protein marker) and GM1 (lipid raft marker).

It has been reported that NF-κB activation in T-cells downstream of TCR engagement strictly occurs in plasma membrane lipid rafts [23], and that Tax also mediates persistent NF-κB activation in the membrane lipid rafts [29,54]. To further characterize the CADM1 and Tax sub cellular localization, we used confocal fluorescence imaging. CADM1 staining was substantially localized in the plasma membrane and cis-Golgi (as determined by Golgi marker GM-130) and Tax was localized in the plasma membrane, cytoplasm and nucleus (consistent with previously published reports [29,55,56] in MT-2 (Fig. 6A), MT-4 (S5A Fig.), and in C8166 cells (S9A Fig.). Next, we co-stained Tax and CADM1 with cholera toxin B labeled with red fluorescence dye, which binds specifically to the sphingolipid-enriched microdomains and found that significant portions of Tax and CADM1 proteins overlapped with GM1 in MT-2 (Fig. 6B), MT-4 (S5B Fig.) and C8166 cells (S9B Fig.). Thus, significant amounts of Tax co-localizes with CADM1 in the lipid rafts.

Since K63-linked polyubiquitination of Tax is critical for IKK complex activation [57], which is initiated in the membrane lipid rafts [29], further experiments were conducted to determine whether Tax undergoes K63-linked polyubiquitination in the membrane lipid raft or in cytoplasmic portion in HTLV-1 transformed cells. We made lysates from MT-2 cells stably expressing lentiviral control shRNA or CADM1 shRNA and subjected these lysates to density gradient ultracentrifugation. Lysates obtained from density gradient ultracentrifugation were split equally into two parts, one was used for immunoprecipitation with anti-Tax and immunoblot with anti-K63-Ubi and anti-Tax, the other was used for immunoprecipitation with anti-CADM1 and immunoblot with known Tax-interacting molecules TAX_1_BP_1_, NEMO, Ubc13 and NRP. As shown in MT-2 (Fig. 6C) and in MT-4 cells (S5C Fig.), Tax underwent K63-linked polyubiquitination only in the lipid raft fractions of control shRNA expressing cells; however, Tax was unable to undergo K63-linked polyubiquitination in CADM1 knockdown cells. Similarly, Tax interacted with TAX_1_BP_1_, NEMO, Ubc13 and NRP in the lipid raft fractions, corresponding to the fractions with the lipid raft markers GM1 and LAT (fractions 4, 5 and 6) from MT-2 and MT-4 cells expressing control shRNA; however, Tax was unable to interact with these molecules upon knockdown of CADM1. Examination of the total and phosphorylated IKKα/β and Tax-associated proteins (TAX_1_BP_1_, NEMO, Ubc13 and NRP) in the lysate fractions from MT-2 and MT-4 cells expressing control shRNA or CADM1 shRNA showed that IKKα/β was robustly phosphorylated only in the lipid raft fractions (fractions 4, 5 and 6) from MT-2 and MT-4 cells expressing control shRNA; however, IKK activation was impaired in the lipid raft fractions (fractions 4, 5 and 6) from MT-2 and MT-4 cells expressing CADM1 shRNA (Figs. 6C and S5C). We also observed that when CADM1 is present in the cytoplasm Tax does not undergo K63-linked polyubiquitination in the cytoplasm, nor does it interact with its associated proteins in the cytoplasm.

We also sought to determine whether Tax and its associated proteins, TAX_1_BP_1_, NEMO, Ubc13 and NRP were localized in the plasma membrane by co-staining with cholera toxin B labeled with red fluorescence dye and confocal fluorescence imaging technique in MT-2 cells. As expected, Tax and its associated proteins were mostly localized in the cytoplasm in MT2 cells and significant portions of Tax and TAX_1_BP_1_, NEMO, Ubc13 and NRP overlapped with GM1, shown in the merged pictures (S8 Fig.). These results strongly suggest that Tax and its associated proteins localized in the membrane lipid rafts of HTLV-1 transformed MT-2 cells.

To determine if K63-linked polyubiquitination of Tax and Tax interaction with its associated molecules occurred in the HTLV-1 transformed Tax expressing MT-2 cells treated with cholesterol-chelating agent, methyl-β-cyclodextrin (MβCD) − a selective cholesterol inhibitor that impairs formation of lipid rafts. Lysates obtained from density gradient ultracentrifugation were split equally in two parts, and immunoprecipitated as described above. As expected K63-linked polyubiquitination of Tax, IKK activation, and Tax interaction with CADM1, TAX_1_BP_1_, NEMO, Ubc13 and NRP were intact in the membrane lipid rafts of control treated (media) MT-2 cells. However, K63-linked polyubiquitination of Tax, IKK activation, and Tax interaction with CADM1, TAX_1_BP_1_, NEMO, Ubc13 and NRP were impaired in the MβCD treated MT-2 cells (S6 Fig.). These results strongly suggest that intact lipid raft is critical for K63-linked polyubiquitination of Tax and Tax interaction with its associated molecules to activate IKK complex in HTLV-1 infected cells.

### CADM1 is not involved in Tax-mediated IKK activation in an *in vitro* cell free system, but is required in intacT-cells

It has been previously demonstrated that K63-linked polyubiquitination of Tax induced by cytosolic factors is sufficient to activate the IKK complex and the first round of IκBα phosphorylation *in vitro* cell-free system. Therefore, we utilized a cell-free system, which lacks plasma membrane associated CADM1, to ascertain whether CADM1 plays any role in Tax-mediated activation of the IKK complex, and the induction of the first round of IκBα phosphorylation and degradation. Cytosolic extracts prepared from Jurkat T-cells, NEMO deficient JM4.5.2 cells, and *Cadm1* wildtype and deficient MEFs were incubated with recombinant Tax (S10A Fig.), and immunoprecipitated with anti-Tax followed by immunoblotting with anti-NEMO, anti-phospho-IKKα/β, anti-IKKα, anti-IKKβ, anti-CADM1, and anti-Tax. Lysates from these reaction mixtures were also examined for Tax-mediated phosphorylation and degradation of IκBα, expression levels of IKKα, IKKβ, NEMO, CADM1, and β-actin. As expected, IKK complex activation, phosphorylation and degradation of IκBα, and Tax-IKK interactions were impaired in NEMO-deficienT-cells. Interestingly, IKK complex activation, phosphorylation and degradation of IκBα, and Tax-NEMO interactions occurred in CADM1-deficient cytosolic extracts similar to Jurkat T-cells and *Cadm1* wildtype extracts (S10B Fig.). We next sought to determine if endogenous CADM1 was required for Tax-NEMO interaction, IKK complex activation, IκBα phosphorylation and degradation in intacT-cells. Lysates from stably expressed lentiviral Tax in Jurkat T-cell, NEMO-deficient JM4.5.2 cells, and *Cadm1* wildtype and deficient MEFs (S10C Fig.), or stably expressing control scrambled shRNA or CADM1 shRNA in HTLV-1 transformed C8166, MT-2, and MT-4 cells (S10D Fig.) were immunoprecipitated with anti-Tax followed by immunoblotting with anti-NEMO, anti-phospho-IKKα/β, anti-IKKα, anti-IKKβ, anti-CADM1, and anti-Tax. Lysates were also examined for Tax-mediated IκBα phosphorylation and degradation. As expected, we observed interactions between Tax and NEMO and IKK complex activation and the first round of IκBα phosphorylation and degradation in Jurkat and CADM1 wildtype MEFs cells; however, the interactions between Tax and NEMO and IKK complex activation and first round of IκBα phosphorylation and degradation were impaired in CADM1-deficienT-cells similar to NEMO-deficienT-cells (S10C-S10D Fig.). These results clearly indicate that the mechanisms of Tax-mediated activation of IKK and the induction of IκBα phosphorylation and degradation in an *in vitro* cell-free system and in intacT-cells are partly different.

### CADM1 is required for Tax to inactivate the A20 complex

**Fig 7:**
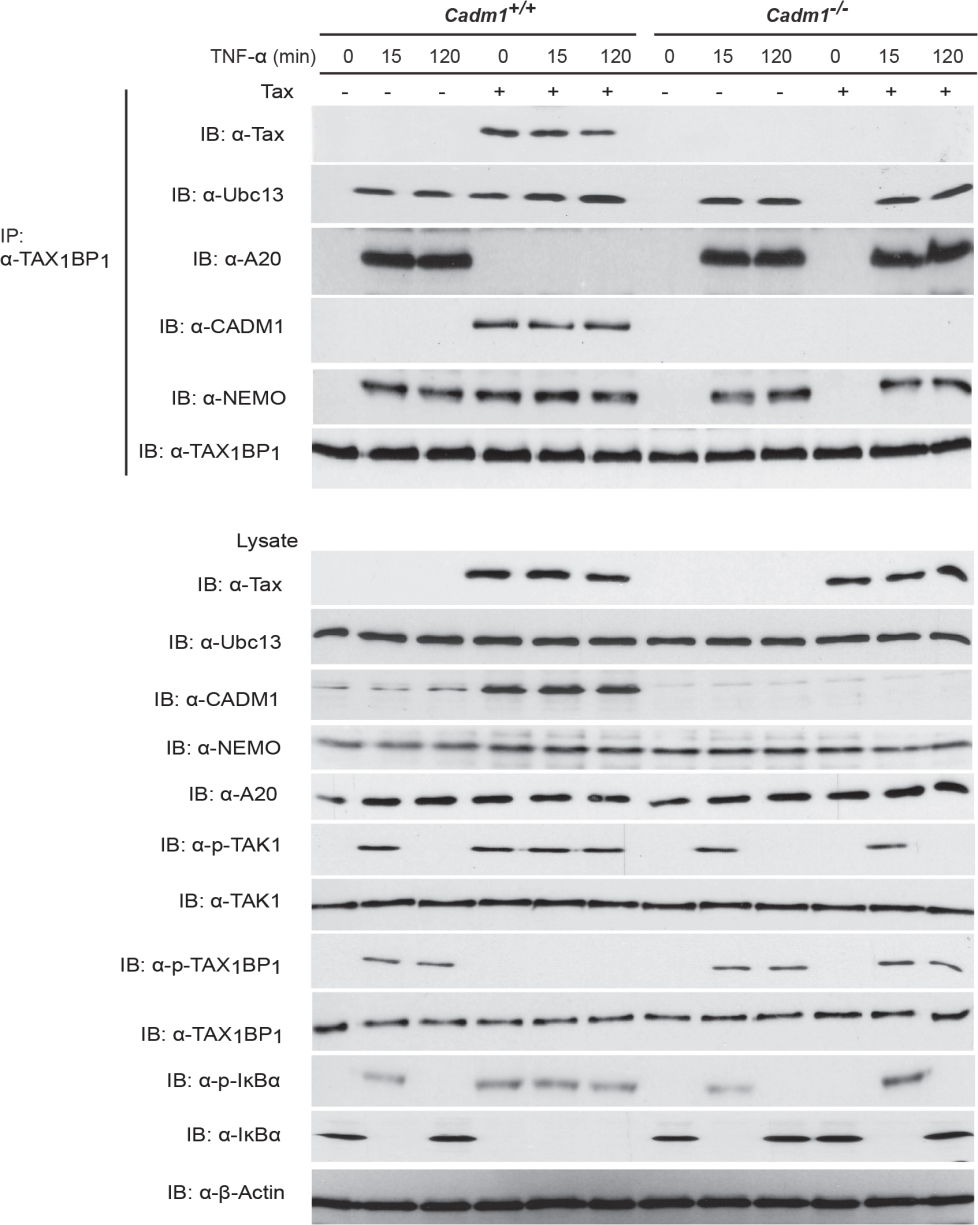
Tax requires CADM1 to inhibit IKKα-mediated phosphorylation of TAX_1_BP_1_ and inactivate the NF-κB inhibitor ubiquitin-editing enzyme A20 complex in the TNFR pathway. Primary *Cadm1* ^*+/+*^ and *Cadm1* ^*−/−*^ MEFs were transduced with Tax-expressing lentiviruses. After 48 hour, cells were treated for up to 120 minutes with TNF-α and lysates were immunoprecipitated with anti-TAX1BP1 followed by immunoblotting with anti-Tax, anti-Ubc13, anti-A20, anti-CADM1, anti-NEMO, and anti-TAX_1_BP_1_ antibodies. Lysates were examined for Tax, Ubc13, TAX_1_BP_1_, phospho-TAX_1_BP_1_, CADM1, NEMO, A20, TAK-1, phospho-TAK1, IκBα, phospho-IκBα, and β-actin expression.

Studies have demonstrated that the TNFR and TLR4/IL-1Rβ-mediated activation of the canonical NF-κB pathway is tightly regulated by the A20 enzyme complex [19,30]. TAX_1_BP_1_ is a critical adaptor molecule for the A20 enzyme complex and is essential for the negative regulation of the canonical NF-κB pathway [19,32,58], thus prompting us to examine whether CADM1 is required for Tax to target TAX_1_BP_1_ phosphorylation and inactivation of the A20 complex. Lentiviruses expressing empty vector or Tax were transduced in *Cadm1* wildtype and deficient MEFs and stimulated with either TNF-α or IL-1β at various times to induce phosphorylation of TAX_1_BP_1_. As expected, TAX1BP1 was inducibly phosphorylated and interacted with A20 (Fig. 7). Transient phosphorylation and degradation of IκBα occurred after 15 minutes in empty vector expressing wildtype *Cadm1* MEFs stimulated with TNF-α or IL-1β; however, Tax completely blocked the phosphorylation of TAX_1_BP_1_ and A20 interaction with TAX_1_BP_1_ and triggered the persistent phosphorylation and degradation of IκBα in wildtype *Cadm1* MEFs treated with TNF-α (Fig. 7) or IL-1β (S4 Fig.). Interestingly, TAX_1_BP_1_ phosphorylation and interaction with A20, and phosphorylation and degradation of IκBα, were normal and transient in empty vector or Tax expressing *Cadm1*-deficient MEFs stimulated with TNF-α (Fig. 7) or IL-1β (S4 Fig.). These results clearly suggest that Tax requires CADM1 to inhibit TAX_1_BP_1_ phosphorylation and inactivation of A20, and to maintain persistent NF-κB activation in HTLV-1 infected T-cells.

## Discussion

The functional activities of CADM1 that have been described to-date include suppression of tumor growth (such as NSCLC), activation of NK or CD8^+^ T cells by serving as a tumor antigen, regulation of cell-cell interactions, and regulation of proper T-cell functions [59–61]. CADM1 expression is frequently down-regulated and its promoter hypermethylated in many human cancers, including lung, prostate, pancreatic, gastric, breast, esophageal and uterine cervix cancer [62,63]. In addition to the hypermethylation, Takai et al. have recently found that miR-214, miR-199, and hypoxia also down-regulate Necl-2 protein expression [64,65]. Restoration of CADM1 expression in NSCLC cells induced apoptosis and inhibited cell proliferation [66]. However, more than a 30-fold upregulation of CADM1 in HTLV-1-associated ATL tumor cells was critical for tumor cell progression and invasion [34]. Morishita et al. have reported that mice receiving CADM1-expressing T-lymphoma cell lines died due to massive tumor metastasis, suggesting that hyper-expression of CADM1 in T-lymphoma cells aggressively promotes leukemia/lymphoma [48]. However, the mechanistic role of CADM1-mediated leukemia/lymphoma cell proliferation and survival is not known. Although ATL cell survival and proliferation is partly dependent on persistent NF-κB activation [67,68] the mechanism of NF-κB activation in ATL cells is poorly understood.

In the current study, we have demonstrated that the HTLV-1 Tax protein induces the aberrant expression of CADM1 through NF-κB and CREB-dependent activation pathways in MEFs, consistent with recent studies by Kim et al. that demonstrated CADM1 mRNA and protein expression in Jurkat T-cells [46]. The findings from our study demonstrate that Tax preferably interacts with the cytoplasmic tail of CADM1 in HTLV-1 transformed T-cell lines. Moreover, Tax requires CADM1 for its K63-linked polyubiquitination, NF-κB activation, and inactivation of the NF-κB negative regulatory A20 complex.

**Fig 8:**
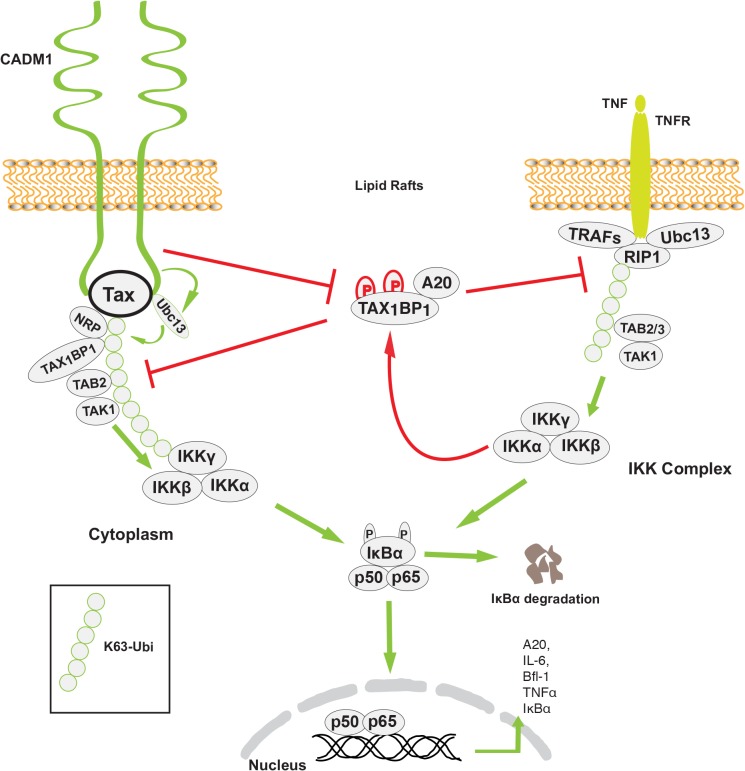
Model of the role of CADM1 in Tax-mediated NF-κB activation. Membrane associated CADM1 recruits Ubc13 on Tax, which causes K63-linked polyubiquitination of Tax, and association of TAX_1_BP_1,_ NRP, and the NEMO/IKK complex with Tax. CADM1 also inhibits IKKα-mediated phosphorylation of TAX_1_BP_1_ and disrupts the NF-κB negative regulator ubiquitin-editing enzyme A20 complex assembly that allows chronic NF-κB activation in HTLV-1 transformed cells.

Previous studies have demonstrated that Tax interactions with Ubc13, NEMO, TAX_1_BP_1_ and NRP are critical for activation of the IKK complex [15,19,53,69]. Our study suggests that CADM1 most likely recruits Ubc13 on Tax, which causes K63-linked polyubiquitination of Tax, and association of ubiquitin binding domain/motif containing NEMO, TAX_1_BP_1_ and NRP proteins to Tax. Stimulus-dependent activation of the IKK complex by TNFR and TCR engagement is generally initiated in the membrane-associated lipid rafts [70]. The upstream signaling molecules for these pathways (RIP1 for TNFR1, and ZAP70 and phosphatidylinositol 3-kinase for TCR) are the key proteins involved in IKK activation in the lipid rafts [71]. A previous study has demonstrated that Tax-mediated chronic NF-κB activation is initiated in lipid raft microdomains in intacT-cells [29]. Tax activates the IKK complex by interacting with its adaptor molecules in the membrane lipid rafts. Interestingly, Tax-mediated IKK complex activation in the membrane lipid rafts occurs in the absence of receptor engagement, suggesting that some lipid raft associated molecules are critical for Tax-mediated IKK complex activation. Another study has shown that cytosolic factors are sufficient to activate initial NF-κB activation in an *in vitro* cell free system, where lipid raft microdomains are absent, and claimed that the first round of NF-κB activation by Tax is critical for the induction of cytokines which are involved in NF-κB activation [57]. Although we also observed Tax-mediated IKK complex activation and the first round of NF-κB activation in an *in vitro* cell free system lacking lipid rafts, Tax failed to activate the IKK complex and the first round of NF-κB in CADM1-deficient intacT-cells. Tax-mediated activation of the IKK complex and the first round of NF-κB in the absence of lipid rafts in an *in vitro* cell free system is possibly due to easy and direct access to cytosolic factors that are normally assembled in response to upstream signals that activate the IKK complex in intacT-cells. We also observed in our study that the loss of Tax-NEMO interactions and Tax-mediated IKK activation in the absence of CADM1 is possibly due to lack of post-translational modifications on Tax. It is also possible that in the absence of CADM1 scaffolding function, Tax-associated molecules are not properly assembled in intact cells. More recent studies from Kim et al. demonstrated that CADM1 interacts with the ζ-chain of TCR to regulate TCR activation and T-cell interactions with APCs [46]. In agreement with this, our results indicate that membrane associated CADM1 is essential for Tax to interact with Ubc13, NEMO, TAX_1_BP_1_ and NRP and to activate the IKK complex in the membrane lipid rafts in the absence of cell stimulation (Figs. 6 and 8).

Previous studies have demonstrated that TAX_1_BP_1_ is a critical adaptor molecule for the NF-κB negative regulatory A20 complex in TNF-α or IL-1β stimulated cells [72]. IKKα-mediated phosphorylation of TAX_1_BP_1_ facilitates A20 complex assembly and subsequent inhibition of NF-κB activation in the TNF-α or IL-1β signaling pathways. Tax inhibits the phosphorylation of TAX_1_BP_1_ by disrupting IKKα and TAX_1_BP_1_ interactions as a mechanism of persistent NF-κB activation in TNF-α or IL-1 stimulated cells. [19]. However the mechanism of IKKα and TAX_1_BP_1_ interactions disruption has remained elusive. Here, we found that the stimulus-dependent phosphorylation of TAX_1_BP_1_ by IKKα is impaired in the absence of CADM1 in Tax expressing cells, suggesting that CADM1 is a crucial molecule for Tax to target the A20 complex (Figs. 7–8). It is possible that CADM1 serves as a critical lipid raft scaffold molecule for Tax, IKKα, TAX_1_BP_1_, and the other adaptor proteins. In future studies we will determine the mechanism of how CADM1 assist Tax to inhibit IKKα-mediated TAX_1_BP_1_ phosphorylation and A20 interaction.

Our results suggest that in HTLV-1 infected T-cell CADM1 does not have tumor-suppressor activity, but rather has gained tumor-promoting activity. It is highly likely that this switch in CADM1 function is triggered by multiple post-translational modifications including phosphorylation, SUMOylation, and ubiquitination. In future studies we will determine the post-translational modification in CADM1 that is responsible for this functional switch.

## Materials and Methods

### Ethics statements

Generation of GZB-Tax transgenic mice was described previously [49], the use of tissues obtained from murine models in this study was carried in strict accordance with the recommendations in the Guide for the Care and Use of Laboratory Animals of the National Institutes of Health. Mice were housed under pathogen-free conditions according to the guidelines of the Division of Comparative Medicine and experiments were approved by the Animal Studies Committee, Washington University School of Medicine under ASC protocol #20100026. Tissues were removed from euthanized animals and placed on ice in PBS during the completion of the necropsy and then frozen on dry ice. Bone marrow was aspirated from long bones, and centrifuged at 2,500 rpm for 5 minutes. The supernatant was aspirated and the cell pellet was frozen on dry ice. The control was an age-matched, sex-matched Tax-negative littermate.

Generation of *Cadm1* ^*−/−*^ mice was described previously [44], animals were housed under specific pathogen free conditions and experiments were carried out in strict accordance with the recommendations in the Guide for the Care and Use of Laboratory Animals of the National Institutes of Health. The protocol was reviewed and approved by the University of Miami Institutional Animal Care and Use Committee (IACUC) (Protocol number: 12–104 RENEWAL 03).

### Biological reagents and cell culture

The human T-cell lymphocytic cell line Jurkat was obtained from ATCC (Manassas, VA) and Tax-expressing HTLV-1-transformed T-cell lines MT-2, MT-4 and C8166 were obtained from the NIH AIDS Reagent Program. The NEMO-deficient JM4.5.2 cell line [69] was a gift from Dr. Sun Sc (The University of Texas MD Anderson, Houston, Texas). Jurkat, JM4.5.2, MT-2, MT-4 and C8166 cells were cultured in RPMI medium (Mediatech, Inc., Herndon, VA) supplemented with 10% fetal bovine serum, 100 U/ml penicillin, and 100 μg/ml streptomycin (Invitrogen, Carlsbad, CA). *Cadm1* ^*+/+*^ and *Cadm1* ^*−/−*^ MEFs were generated using a standard procedure [73]. Briefly, *Cadm1* heterozygous mice described previously [44] were mated and E12.5 embryos were dissected free of surrounding tissues, washed in PBS (phosphate-buffered saline), and the heads and livers removed. The tissue was placed in 3 ml of 0.25% trypsin/EDTA and disrupted by forcing through a 6 cc syringe followed by vigorous pipetting, and the contents were transferred into a T25 tissue culture flask before placing in a tissue culture incubator at 37^0^ C and 5% CO_2_ for 5 minutes. *Cadm1* ^*+/+*^ and *Cadm1* ^*−/−*^ MEFs were cultured in complete DMEM medium (Mediatech; Manassas, VA) containing 20% fetal bovine serum, heat inactivated, sterile-filtered (Sigma-Aldrich), L-glutamine, 1x penicillin-streptomycin (Invitrogen/Life Technologies). The plasmids pCAGI-Puro-FLAG-CADM1, pCAGI-Puro-FLAG-CADM1-ΔCP (deleting the cytoplasmic tail, aa 404–445), pCAGI-Puro-FLAG-CADM1-ΔEC (deleting the extracellular region, aa 1–362), pCAGI-Puro-FLAG-CADM1-ΔFERM (deleting the FERM domain-binding motif, aa 401–413), and pCAGI-Puro-FLAG- CADM1-ΔPDZ-BM (deleting the PDZ domain-binding motif, aa 442–445) were described previously [41]. The pCMV4-Tax, Tax M22, Tax M47 and NF-κB-TATA luciferase constructs have been described previously [32]. Tax M22 and M47 were constructed by replacing G137A and L138S, and L319R and L320S amino acid substitutions using a QuikChange site-directed mutagenesis kit (Stratagene, La Jolla, CA). All mutations were confirmed by DNA sequencing. The following antibodies were used in this study: anti-β-actin (Abcam), anti-TAX_1_BP_1_ (Abcam), anti-A20 (BD Biosciences Pharmingen and EMD Millipore), anti-phospho-TAX_1_BP_1_ described previously [19] was a gift from Dr. Edward Harhaj (Johns Hopkins School of Medicine), anti-ERK1/2, anti-phospho-TAK1, anti-phospho-IκBα, anti-phospho-IKKα/β (Cell Signaling), anti-CADM1 (MBL International Corporation), anti-CADM1, anti-TAK-1, anti-NEMO, anti-IKKα, anti-IKKβ, anti-IκBα (Santa Cruz Biotechnology), anti-Flag (Sigma), anti-NRP (Cayman Chemical), anti-LAT (Upstate Biotechnology), anti-Ubc13 (clone 4E11; Invitrogen), and antibody specific for ubiquitin Lys63 (HWA4C4; Millipore). Anti-Tax [31] was prepared from a Tax hybridoma (168B17-46-34) from the AIDS Research and Reference Program of the National Institute of Allergy and Infectious Diseases (US National Institutes of Health). Recombinant TNF and IL-1 were purchased from R&D Systems. The Optiprep was from Axis-Shield (Oslo, Norway).

### Lentiviral particle production and targeT-cell infection

To investigate the role of CADM1 in Tax-mediated NF-κB activation, a CADM1-specific shRNA construct was used to knockdown CADM1 expression. HEK 293-T-cells in 6-well culture plates were transfected with 1 μg of control scrambled shRNA or CADM1 shRNA with 2 μg of packaging plasmids (OriGene Technologies) containing puromycin selection marker using FuGENE 6 (Roche). Seventy-two hours post-transfection, the supernatants were collected and concentrated by ultracentrifugation and the pellets were resuspended in ice-cold PBS. Viral stocks were used to infect Jurkat T-cells, MEFs and HTLV-1 transformed (C8166, MT-2, and MT-4) cells and selected with puromycin. To overexpress Tax in MEFs and Jurkat T-cells, pCMV-Tax was used as a template for PCR-mediated cloning into the pDUET-GFP-hygromycin and pCDH-Cuo-MCS-EFI-GFP-T2A-puro lentiviral vector. Lentiviruses expressing Tax or control (GFP) empty vector were generated as described above. MEFs or Jurkat T-cells were infected with lentiviruses and selected with puromycin or hygromycin after 48 hours.

### Transfections and luciferase assays

Transient transfections in MEFs and Jurkat T-cells were performed using FuGENE HD (Roche) according to the manufacturer's instructions. For luciferase assays, cells were harvested after 36–48 hours post-transfection and cell lysates were prepared in 1× Passive Lysis Buffer (Promega). Luciferase activity was assayed using the Dual Luciferase Assay system according to the manufacturer's instructions (Promega). All luciferase transfections included the *Renilla* luciferase reporter pRL-tk to normalize for transfection efficiency. Error bars indicate the standard error of the mean (s.e.m.) of triplicate samples from a representative experiment.

### RT-PCR

RT-PCR was done as described previously [15]. Total RNA was obtained from cells by using an RNeasy kit (QIAGEN, Valencia, CA) and converted to cDNA using a first-strand cDNA synthesis kit (Roche). The following sets of primers were used to amplify gene products for PCRs: glyceraldehyde-3-phosphate dehydrogenase (GAPDH) (263 bp) forward-5′-CCA CAG TCC ATG CCA TCA C-3’ and reverse-5′-GCT TCA CCA CCT TCT TGA TG-3’; Tax (429 bp) forward-5′-CGG ATA CCC AGT CTA CGT C-3’ and reverse-5′-GAG GTA CAT GCA GAC AAC GG-3’; IL-6 (460 bp) forward-5′-GAC TTC ACA GAG GAT ACC ACT C-3’ and reverse-5′-GTC CTT AGC CAC TCC TTC TG-3’; A20 (560 bp) forward-5′-GAC AGA AGT GTC CAG GCT TC-3’ and reverse-5′-GTG CTG GCT GTC ATA GCC TAG-3’; and CADM1 (477 bp) forward-5’-GAT GAT CGA TAT CCA GAA AGA CAC-3’ and reverse-5’-GTT TTG TTT AGG TTA TTG ATG AAC AG. Bfl-1 (394) forward-5’-TAC AGG TAC CCG CCT TTG AG-3’ and reverse-5’-TCT TCC CAA CCT CCA TTC TG-3’. Human SOCS1 (400) forward-5’- GACGCCTGCGGATTCTACTG-3’ and reverse-5’-GGAAGGAGCTCAGGTAGTCG-3’, mouse SOCS1 (453) forward-5’-GACACTCACTTCCGCACCTTCC-3’ and reverse-5’-GTCACGGAGTACCGGGTTAAGAG-3’.

### Lipid raft isolation

Lipid raft fraction analysis was carried out as described previously [29]. Briefly, HTLV-1 transformed MT-2 cells were lysed in 2 ml of extraction buffer (20 mM Tris-Cl, pH 7.4, 150 mM NaCl, 1 mM EDTA, 1% Triton X-100 plus protease inhibitor cocktail). Lysates were combined with a 60% Optiprep solution to yield 40% and placed at the bottom of the ultracentrifuge tube followed by overlaying with an equal volume (4 ml) of discontinuous 30% and 5% OptiPrep Density Gradient medium. Samples were centrifuged at 100,000 × g for 4 hours at 4°C in an SW41 rotor. 1 ml of each fraction from the top to bottom was collected and equal volumes of each fraction were loaded onto SDS-PAGE gels. For the depletion of plasma and intracellular membrane cholesterol by MβCD in MT-2 cells cultured in RPMI medium supplemented with fetal bovine serum (10%) and penicillin-streptomycin (1%), were treated with or without 10 mM MβCD and incubated at 37°C for 45 min. Followed by this step lysates were subjected to density gradient ultracentrifugation for lipid raft fractionation analysis.

### Purification of the recombinant Tax

Tax was expressed from the pTaxH_6_ expression plasmid and purified as previously described [74,75].

### Preparation of cytoplasmic extracts (S100) and cell free assay for IKK activation

Cytoplasmic extract from Jurkat, NEMO-deficient JM4.5.2 (Harhaj et al. 2000 Oncogene 19:1448–56), *Cadm1* ^*+/+*^ and *Cadm1* ^*−/−*^ MEFs were prepared as described earlier [57]. Briefly, cytoplasmic extracts were prepared by lysing the cells in a hypotonic buffer (10 mM Tris HCl (pH 7.5), 1.5 mM MgCl2, 10 mM KCl, 0.5 mM dithiothreitol (DTT) and a protease inhibitor cocktail (Roche)) and homogenized using a Dounce homogenizer. Lysates were placed on ice for another 10 minutes. After centrifugation at 100,000g for 1 hour at 4°C the supernatant (S100) was collected. Recombinant Tax was incubated in cytosolic extract (10 mg/ml) containing ATP buffer (50 mM Tris HCl (pH 7.5), 5 mM MgCl2, 2 mM ATP, 5 mM NaF, 20 mM β-glycerophosphate, 1 mM Na3VO4 and a protease inhibitor cocktail). After incubation at 30° C for 1 hour, the reaction mixtures were subjected to western blotting.

### Co-IP and ubiquitination assays

Ubiquitination assays were performed essentially as described previously [19,72]. Briefly, MEFs, Jurkat or HTLV-1 infected (C8166, MT-2 and MT-4) cells were lysed in RIPA buffer and immunoprecipitated with Tax antibody, and eluted with 1% SDS, diluted in lysis buffer, re-immunoprecipitated with Tax antibody, and detected by immunoblotting with antibodies to K63-Ubi or Tax. Similarly, the fractions obtained from density gradient ultracentrifugation or cells lysed in RIPA buffer were immunoprecipitated with specific antibodies. Immunoprecipitates were washed three times with respective buffers. Immunoblotting was performed with the indicated antibodies for co-IPs.

### Immunoblotting

Immunoblotting was performed as described earlier [32,76]. Whole-cell lysates were resolved by SDS–PAGE, transferred to nitrocellulose membranes, blocked in 5% milk, incubated with the indicated primary and secondary antibodies and then detected with Western Lightning Enhanced Chemiluminescence reagent (Perkin Elmer).

### Confocal microscopy

The cells were seeded onto 12-mm poly-L-lysine-coated coverslips (BD Biosciences, Bedford, MA) and were briefly centrifuged prior to fixation. The cells were washed three times with PBS and fixed in 4% paraformaldehyde for 15 minutes at room temperature. The fixed cells were permeabilized with PBS containing 0.2% Triton X-100, and nonspecific binding was prevented by a 1 hour incubation in SuperBlock buffer (Thermo Scientific) followed by staining with primary antibodies: mouse anti-Tax, rabbit anti-CADM1 (Santa-Cruuz biotechnology), anti-Ubc13, anti-NEMO, anti-NRP (Fisher Scientific), anti-TAX1BP1, anti-GM130 (Abcam), and chicken anti-CADM1 (MBL International Corporation), diluted in PBS containing 1% BSA and incubated for 2 hours followed by five washes with PBS containing 1% BSA. Secondary antibodies: Alexa Fluor 555- donkey anti-mouse or Alexa Fluor 647- donkey anti-mouse IgG (for Tax), Alexa Fluor 488-donkey anti-rabbit IgG (for CADM1, TAX1BP1, Ubc13, NEMO, NRP), Alexa Fluor 647- donkey anti-rabbit IgG (for Golgi-130), Alexa Fluor 555-conjugated cholera toxin subunit B (Invitrogen), and Cy2 donkey anti-Chicken IgG, (for CADM1) Jackson ImmunoResearch) were incubated for 45 min followed by four washes with PBS. The cells were then incubated with DAPI 500ng/ml (Sigma). After washing three times with PBS, the coverslips were mounted onto the glass slides with ProLong Gold anti-fade reagent (Invitrogen) and then observed under SP5 confocal microscope (Leica).

### EMSA

Nuclear extracts were prepared from HTLV-1 transformed (C8166, MT-2, and MT-2), and MEFs cells. The NF-κB electrophoretic mobility shift assay (EMSA) was done as described previously [15,32]. The Oct-1 EMSA probe was generated by annealing the following oligonucleotides: forward 5′-TGTCGAATGCAAATCACTAGAA-3’ and reverse 5′-TTCTAGTGATTTGCATTCGACA-3’. The annealed oligonucleotides were labeled with (^32^P) dTTP in a fill-in reaction with Klenow fragment (Promega). Nuclear extract (4 μg) was incubated with buffer containing 1 mM dithiothreitol, 1 μg poly(dI-dC), dialysis buffer (25 mM HEPES, pH 7.9, 10% glycerol, 100 mM KCl, and 0.1 mM EDTA), and ^32^P-labeled probe for 15 minutes. The reaction was terminated by the addition of 5× loading dye, and the reaction mixture was run on 5% polyacrylamide gels in 0.25× Tris-borate-EDTA buffer, dried under vacuum, and subjected to autoradiography.

## Supporting Information

S1 Fig Tax mediated induction of CADM1 mRNA. (A) MEFs, and (B) Jurkat T-cells were transduced with lentiviruses expressing control GFP, wildtype Tax, or its mutants (M22 and M47). After 48 hours, RNA was prepared and subjected to RT-PCR for CADM1, Tax, and GAPDH expression. (C) Expression of CADM1, Tax, and β-actin in Jurkat T-cells and Tax expressing in HTLV-1 transformed (C8166, MT-2 and MT-4) cells.
(TIF)

S2 Fig CADM1 is required for Tax to interact with Ubc13. (A) Immunoblot analyses of CADM1 protein expression in HTLV-1 transformed MT-2 cells after transduction with four lentiviruses expressing different shRNAs targeting distinct sequences of the CADM1 transcript. (B) Lysates from HTLV-1 transformed C8166, MT-2 and MT-4 cells stably expressing control scrambled shRNA or CADM1 shRNA were immunoprecipitated with anti-Tax1BP1, followed by immunoblotting with anti-Ubc13, anti-Tax, anti-CADM1, anti-TAX_1_BP_1_, anti-NEMO and anti-NRP antibodies. Lysates were also examined for Ubc13, Tax, CADM1, TAX_1_BP_1_, NEMO and NRP expression using respective antibodies. (C) Lysates from primary *Cadm1* ^*+/+*^ and *Cadm1* ^*−/−*^ MEFs transduced with Tax-expressing lentiviruses were immunoprecipitated with anti-Tax1BP1 followed by immunoblotting with anti-Ubc13, anti-Tax, anti-CADM1 anti-TAX_1_BP_1_, anti-NEMO and anti-NRP antibodies. Lysates were also examined for Ubc13, Tax, CADM1, TAX_1_BP_1_, NEMO and NRP expression using respective antibodies.
(TIF)

S3 Fig Tax requires CADM1 to activate the non-canonical NF-κB pathway. (A) Jurkat T-cells stably expressing control scrambled shRNA or CADM1 shRNA were transduced with lentiviral control GFP or Tax expressing lentiviruses. After 48 hours, lysates were immunoblotted with anti-CADM1, anti-Tax, anti-p100, and anti-β-actin antibodies. (B) Lysates from primary *Cadm1* ^*+/+*^ and *Cadm1* ^*−/−*^ MEFs transduced with lentiviral control GFP or Tax expressing lentiviruses were immunoblotted with anti-CADM1, anti-Tax, anti-p100, and anti-β-actin antibodies.
(TIF)

S4 Fig Tax requires CADM1 to inhibit IKKα-mediated phosphorylation of TAX_1_BP_1_ and inactivate the NF-κB inhibitor ubiquitin-editing enzyme A20 complex in the IL-1βR pathway. Primary *Cadm1* ^*+/+*^ and *Cadm1* ^*−/−*^ MEFs were transduced with Tax-expressing lentiviruses. After 48 hours, cells were treated for 0–120 minutes with IL-1β and lysates were immunoprecipitated with anti-TAX1BP1 followed by immunoblotting with anti-Tax, anti-Ubc13, anti-A20, anti-CADM1, anti-NEMO, and anti-TAX_1_BP_1_ antibodies. Lysates were also examined for Tax, Ubc13, TAX_1_BP_1_, phospho-TAX_1_BP_1_, CADM1, NEMO, A20, TAK-1, phospho-TAK1, IκBα, phospho-IκBα, and β-actin expression.
(TIF)

S5 Fig Membrane associated CADM1 mediates K63-linked polyubiquitination of Tax and links Tax adaptor proteins in the lipid rafts of MT-4 cells. (A) MT-4 cells were stained with DAPI, anti-Tax, anti-CADM1, and anti-GM-130, and subjected to confocal microscopy. (B) MT-4 cells were stained with DAPI, anti-Tax, anti-CADM1, and cholera toxin B conjugated with red fluorescence to detect GM-1 and subjected to confocal microscopy. (C) Lipid raft fractionations from MT-4 cells stably expressing control scrambled shRNA or CADM1 shRNA were split into half and subjected to immunoprecipitation with either anti-Tax or anti-CADM1. Samples immunoprecipitated with anti-Tax were immunoblotted with anti-K63-ubi and anti-Tax. Samples immunoprecipitated with anti-CADM1 were immunoblotted with anti-CADM1, anti-TAX_1_BP_1_, anti-Tax, anti-NEMO, anti-Ubc13, and anti-NRP antibodies. Lysates from lipid rafts fractions were examined for Tax, phospho-IKKα/β, total IKKα, IKKβ, NEMO, CADM1, Ubc13, TAX_1_BP_1_, NRP, ERK1 (marker for soluble fractions), LAT (lipid raft protein marker), and GM1 (lipid raft marker).
(TIF)

S6 Fig Disruption of lipid rafts impairs CADM1 and Tax interaction and Tax K63-linked polyubiquitination. Lipid raft fractionations of MT-2 cells pretreated with MβCD were split into half and subjected to immunoprecipitation with either anti-Tax or anti-CADM1. Samples immunoprecipitated with anti-Tax were immunoblotted with anti-K63-ubi and anti-Tax, and samples immunoprecipitated with anti-CADM1 were immunoblotted with anti-CADM1, anti-TAX_1_BP_1_, anti-Tax, anti-NEMO, anti-Ubc13 and anti-NRP antibodies. Lysates from lipid rafts fractions were examined for Tax, phospho-IKKα/β, total IKKα, IKKβ, NEMO, CADM1, Ubc13, TAX_1_BP_1_, NRP, ERK1 (marker for soluble fractions), LAT (lipid raft protein marker) and GM1 (lipid raft marker).
(TIF)

S7 Fig Tax induces CADM1 expression via NF-κB and CREB. CADM1 expression in lentiviral-transduced empty vector wildtype Tax, Tax single mutants (M22) or (M47), and Tax double mutants (M22 and M47) in primary MEFs was analyzed with anti-CADM1, anti-SOCS1, anti-Tax, and β-actin antibodies.
(TIF)

S8 Fig Lipid raft presence of Tax, Ubc13, TAX1BP1, NEMO, and NRP in MT-2 cells by fluorescence imaging analysis. MT-2 cells were co-stained with DAPI, anti-Tax, cholera toxin B conjugated with red fluorescence to detect GM-1, anti-Ubc13 (A), anti-TAX_1_BP_1_ (B), anti-NEMO (C), and anti-NRP (D) and subjected to confocal microscopy.
(TIF)

S9 Fig Localization of Tax and CADM1 in C8166 cells. (A) C8166 cells were stained with DAPI, anti-Tax, anti-CADM1, and anti-GM-130, and subjected to confocal microscopy. (B) C8166 cells were stained with DAPI, anti-Tax, anti-CADM1, and cholera toxin B conjugated with red fluorescence to detect GM-1, and subjected to confocal microscopy.
(TIF)

S10 Fig CADM1 is not involved in Tax-mediated IKK activation in an *in vitro* cell free system, but is required in intacT-cells. (A) Purified recombinant Tax was analysed by SDS-PAGE and staining with Coomassie brilliant blue. The positions of molecular mass markers (lanes M) (in kilodaltons) are indicated to the left of the gel. An arrow indicates the Tax protein. (B) Induction of IκBα phosphorylation and degradation by recombinant Tax in an *in vitro* cell free system. Cytosolic extracts (10 mg/ml) from either Jurkat, JM4.5.2, *Cadm1* ^*+/+*^ or *Cadm1* ^*−/−*^ cells were incubated with recombinant Tax (250 ng) and ATP (2 nM) at 30°C for 1 hour. Reaction mixture was immunoprecipitated with anti-Tax followed by immunoblotting with anti-NEMO, anti-phospho-IKKα/β, anti-IKKα, anti-IKKβ, anti-CADM1, and anti-Tax. Lysates from these reaction mixtures were further examined for Tax-mediated phosphorylation and degradation of IκBα, expression levels of IKKα, IKKβ, NEMO, CADM1, and β-actin proteins in cytosolic extracts. CADM1 is indispensable for Tax-NEMO binding and Tax-mediated IKK activation, and to induce the first round of IκBα phosphorylation and degradation in intacT-cells. (C) IP-westerns of lysates from lentiviral expressing Tax in either Jurkat, JM4.5.2, *Cadm1* ^*+/+*^ or *Cadm1* ^*−/−*^ cells, (D) stably expressing control scrambled shRNA or CADM1 shRNA in Tax expressing HTLV-1 transformed (C1866, MT-2 and MT-4) cell lines, assessed after immunoprecipitation with anti-Tax and immunoblot with anti-NEMO, anti-phospho-IKKα/β, anti-IKKα, anti-IKKβ, anti-CADM1, and anti-Tax. Below (Lysates), immunoblot analysis of total cell lysates with antibodies along left margins with anti-Tax, anti-NEMO, anti-CADM1, anti-p-IκBα anti-IκBα, and anti-β-actin.
(TIF)
