# Supplementary figures and images for: Human T-Cell Leukemia Virus Type 1 (HTLV-1) Tax Requires CADM1/TSLC1 for Inactivation of the NF-κB Inhibitor A20 and Constitutive NF-κB Signaling
Source: PLoS Pathog. 2015 Mar 16;11(3):e1004721. doi: 10.1371/journal.ppat.1004721 (PMC4361615; doi:10.1371/journal.ppat.1004721)

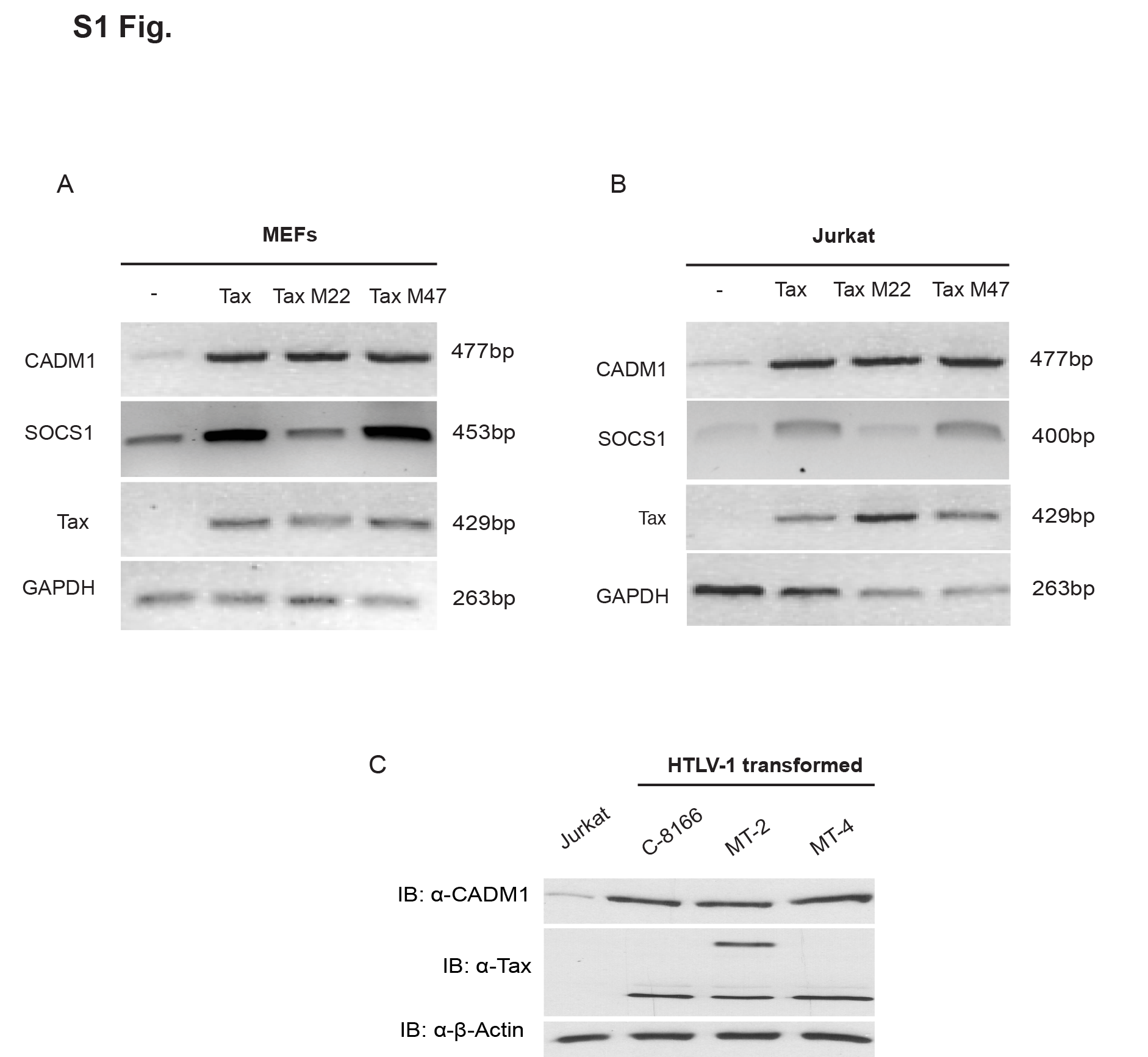

Supplement: S1 Fig — (A) MEFs, and (B) Jurkat T-cells were transduced with lentiviruses expressing control GFP, wildtype Tax, or its mutants (M22 and M47). After 48 hours, RNA was prepared and subjected to RT-PCR for CADM1, Tax, and GAPDH expression. (C) Expression of CADM1, Tax, and β-actin in Jurkat T-cells and Tax expressing in HTLV-1 transformed (C8166, MT-2 and MT-4) cells. (TIF) [file ppat.1004721.s001.tif]

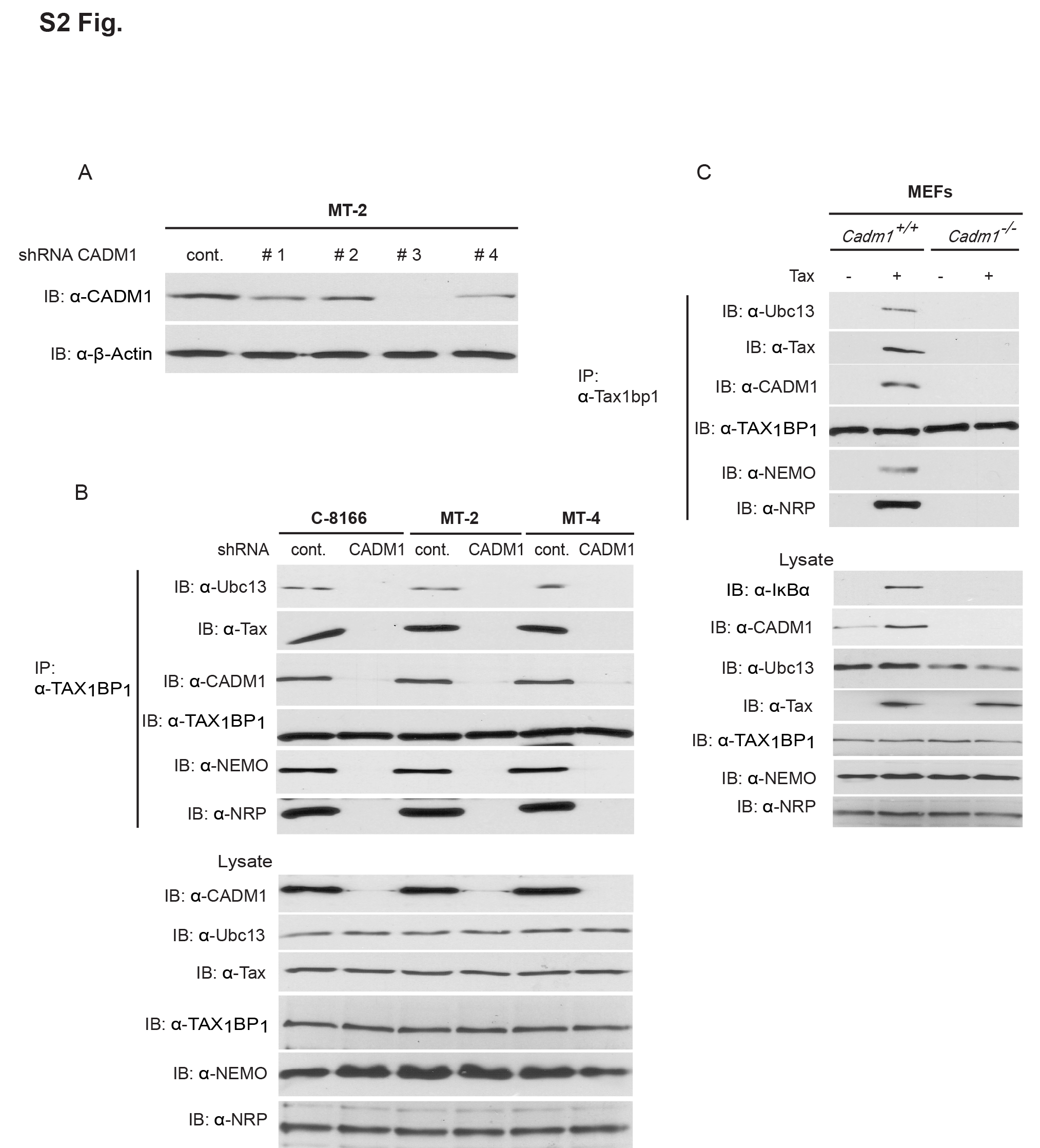

Supplement: S2 Fig — (A) Immunoblot analyses of CADM1 protein expression in HTLV-1 transformed MT-2 cells after transduction with four lentiviruses expressing different shRNAs targeting distinct sequences of the CADM1 transcript. (B) Lysates from HTLV-1 transformed C8166, MT-2 and MT-4 cells stably expressing control scrambled shRNA or CADM1 shRNA were immunoprecipitated with anti-Tax1BP1, followed by immunoblotting with anti-Ubc13, anti-Tax, anti-CADM1, anti-TAX1BP1, anti-NEMO and anti-NRP antibodies. Lysates were also examined for Ubc13, Tax, CADM1, TAX1BP1, NEMO and NRP expression using respective antibodies. (C) Lysates from primary Cadm1 +/+ and Cadm1 −/− MEFs transduced with Tax-expressing lentiviruses were immunoprecipitated with anti-Tax1BP1 followed by immunoblotting with anti-Ubc13, anti-Tax, anti-CADM1 anti-TAX1BP1, anti-NEMO and anti-NRP antibodies. Lysates were also examined for Ubc13, Tax, CADM1, TAX1BP1, NEMO and NRP expression using respective antibodies. (TIF) [file ppat.1004721.s002.tif]

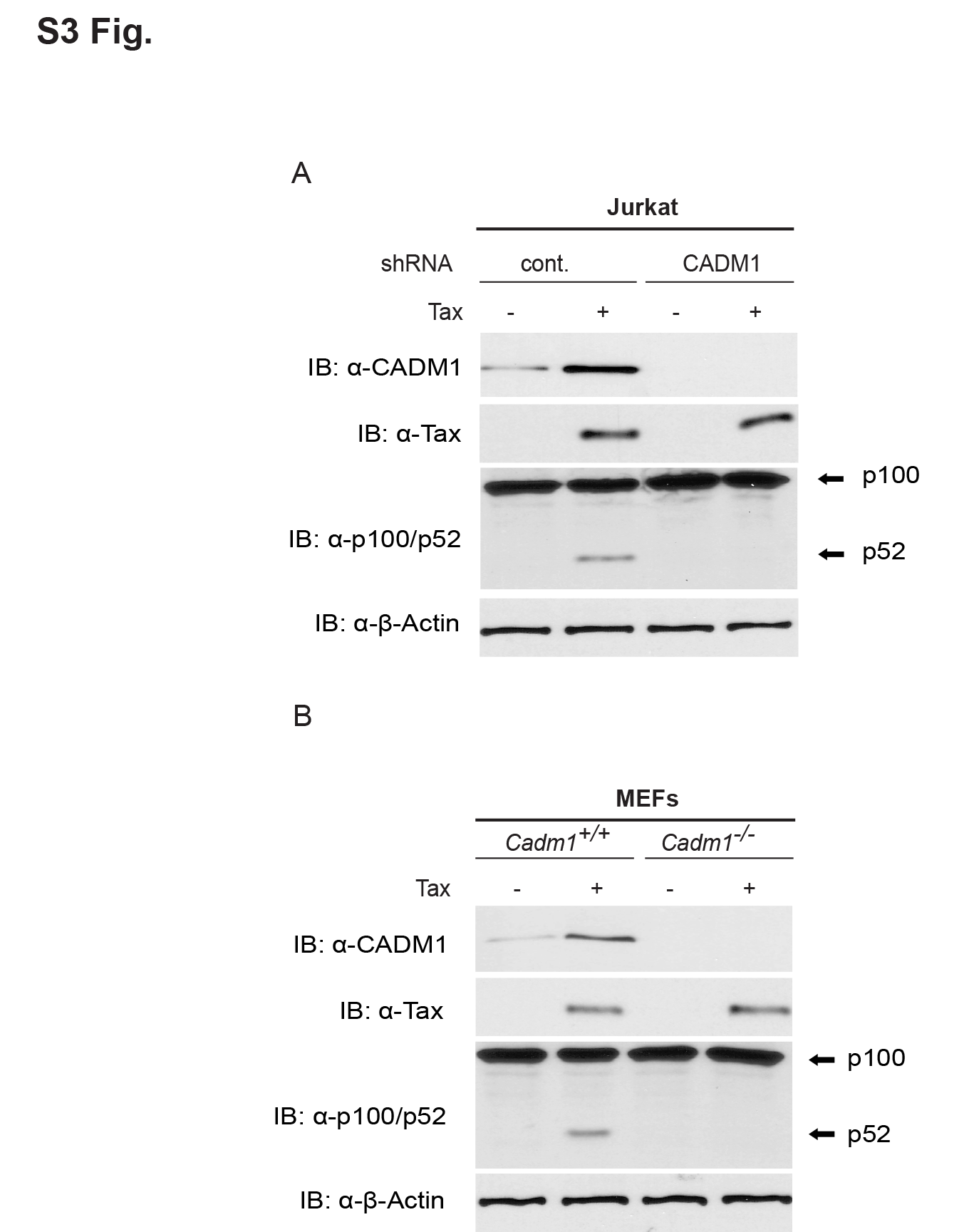

Supplement: S3 Fig — (A) Jurkat T-cells stably expressing control scrambled shRNA or CADM1 shRNA were transduced with lentiviral control GFP or Tax expressing lentiviruses. After 48 hours, lysates were immunoblotted with anti-CADM1, anti-Tax, anti-p100, and anti-β-actin antibodies. (B) Lysates from primary Cadm1 +/+ and Cadm1 −/− MEFs transduced with lentiviral control GFP or Tax expressing lentiviruses were immunoblotted with anti-CADM1, anti-Tax, anti-p100, and anti-β-actin antibodies. (TIF) [file ppat.1004721.s003.tif]

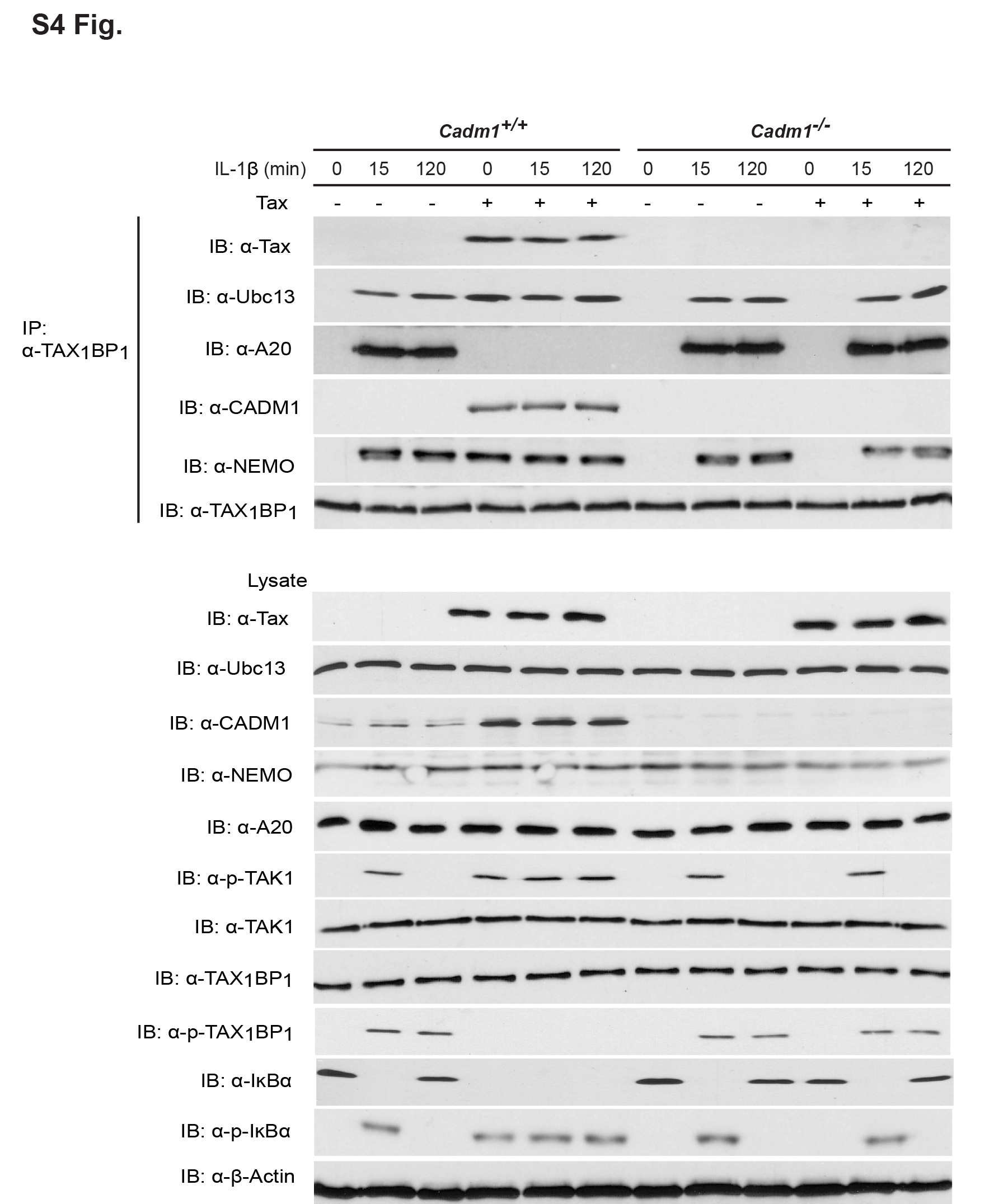

Supplement: S4 Fig — Primary Cadm1 +/+ and Cadm1 −/− MEFs were transduced with Tax-expressing lentiviruses. After 48 hours, cells were treated for 0–120 minutes with IL-1β and lysates were immunoprecipitated with anti-TAX1BP1 followed by immunoblotting with anti-Tax, anti-Ubc13, anti-A20, anti-CADM1, anti-NEMO, and anti-TAX1BP1 antibodies. Lysates were also examined for Tax, Ubc13, TAX1BP1, phospho-TAX1BP1, CADM1, NEMO, A20, TAK-1, phospho-TAK1, IκBα, phospho-IκBα, and β-actin expression. (TIF) [file ppat.1004721.s004.tif]

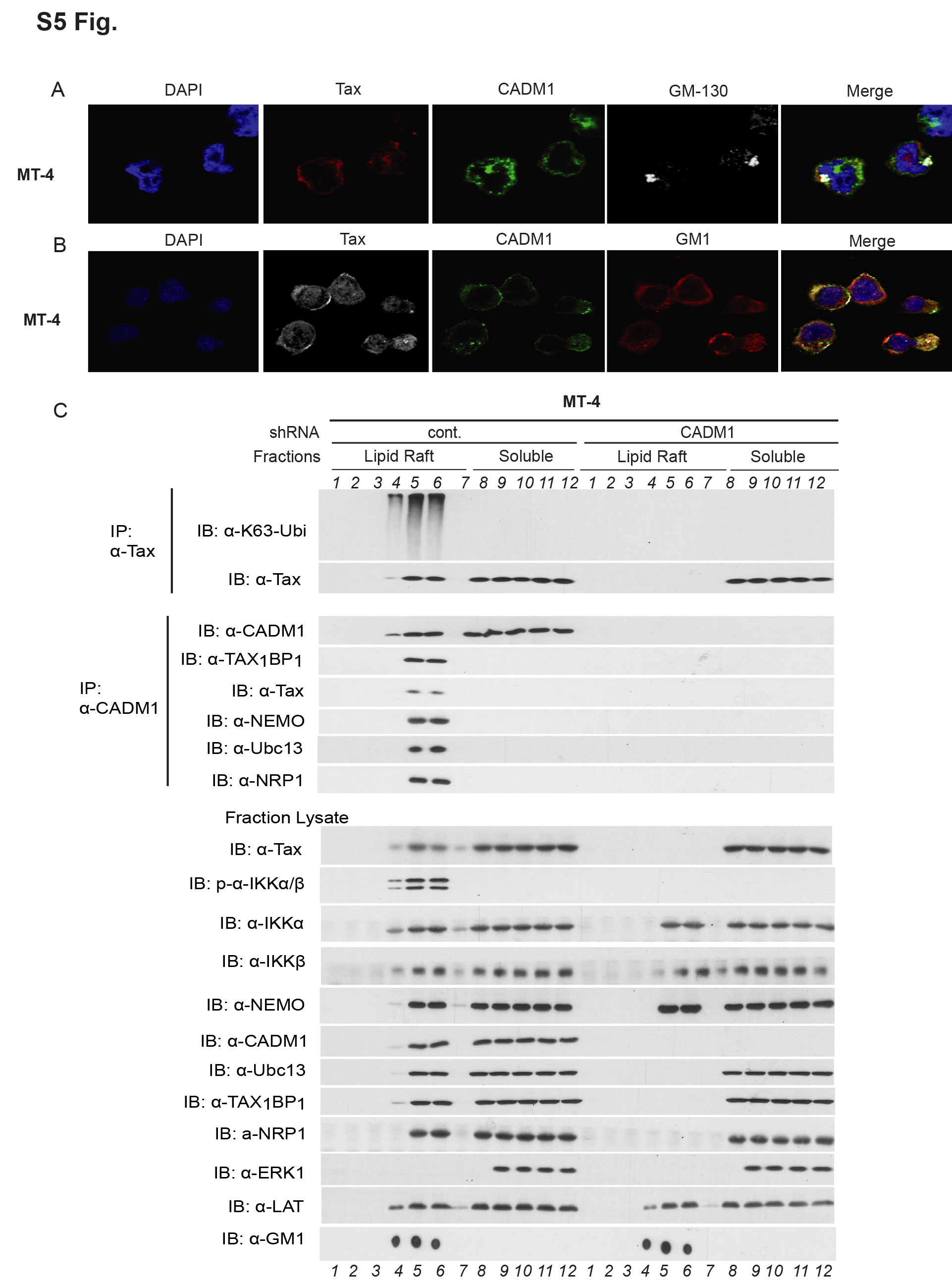

Supplement: S5 Fig — (A) MT-4 cells were stained with DAPI, anti-Tax, anti-CADM1, and anti-GM-130, and subjected to confocal microscopy. (B) MT-4 cells were stained with DAPI, anti-Tax, anti-CADM1, and cholera toxin B conjugated with red fluorescence to detect GM-1 and subjected to confocal microscopy. (C) Lipid raft fractionations from MT-4 cells stably expressing control scrambled shRNA or CADM1 shRNA were split into half and subjected to immunoprecipitation with either anti-Tax or anti-CADM1. Samples immunoprecipitated with anti-Tax were immunoblotted with anti-K63-ubi and anti-Tax. Samples immunoprecipitated with anti-CADM1 were immunoblotted with anti-CADM1, anti-TAX1BP1, anti-Tax, anti-NEMO, anti-Ubc13, and anti-NRP antibodies. Lysates from lipid rafts fractions were examined for Tax, phospho-IKKα/β, total IKKα, IKKβ, NEMO, CADM1, Ubc13, TAX1BP1, NRP, ERK1 (marker for soluble fractions), LAT (lipid raft protein marker), and GM1 (lipid raft marker). (TIF) [file ppat.1004721.s005.tif]

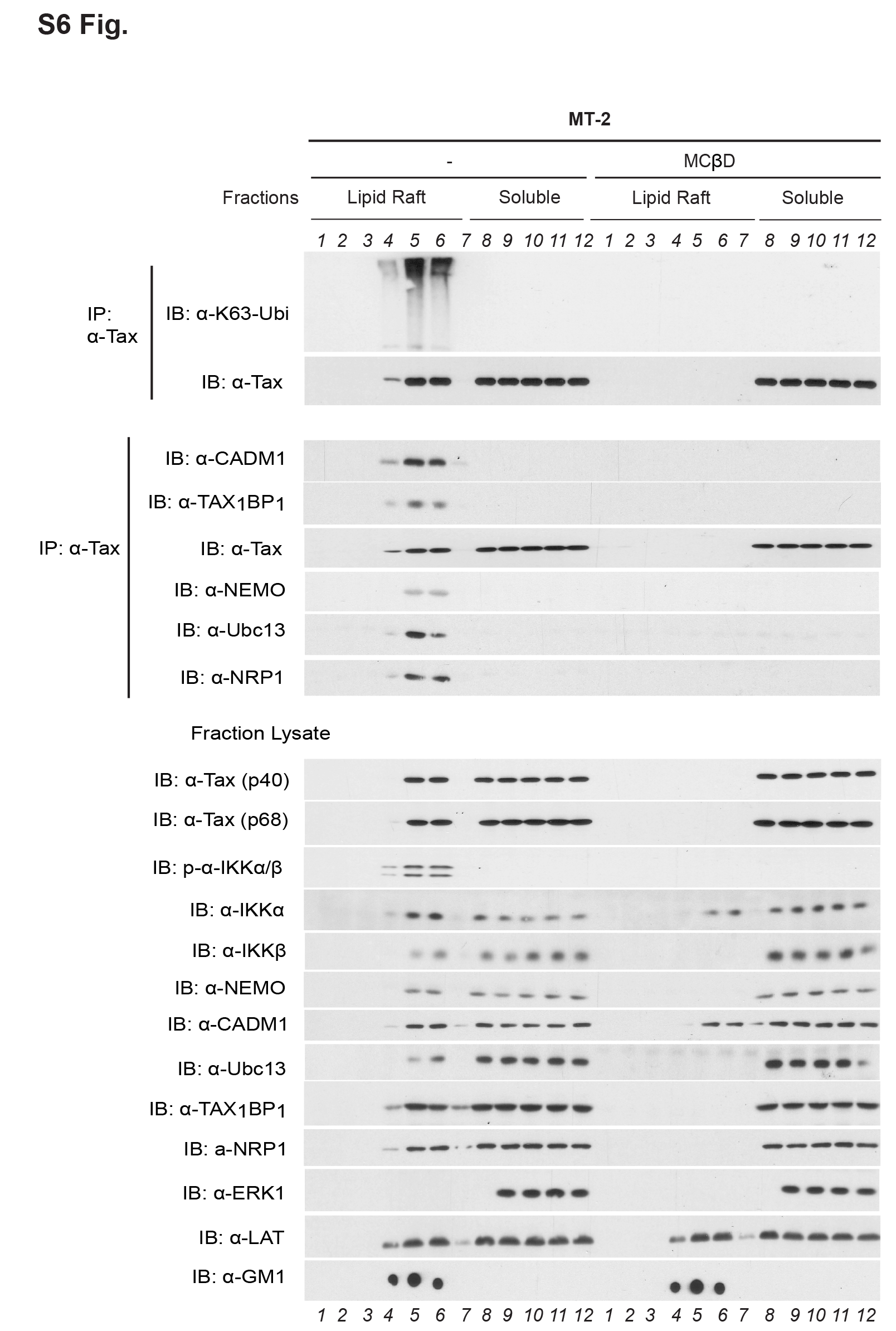

Supplement: S6 Fig — Lipid raft fractionations of MT-2 cells pretreated with MβCD were split into half and subjected to immunoprecipitation with either anti-Tax or anti-CADM1. Samples immunoprecipitated with anti-Tax were immunoblotted with anti-K63-ubi and anti-Tax, and samples immunoprecipitated with anti-CADM1 were immunoblotted with anti-CADM1, anti-TAX1BP1, anti-Tax, anti-NEMO, anti-Ubc13 and anti-NRP antibodies. Lysates from lipid rafts fractions were examined for Tax, phospho-IKKα/β, total IKKα, IKKβ, NEMO, CADM1, Ubc13, TAX1BP1, NRP, ERK1 (marker for soluble fractions), LAT (lipid raft protein marker) and GM1 (lipid raft marker). (TIF) [file ppat.1004721.s006.tif]

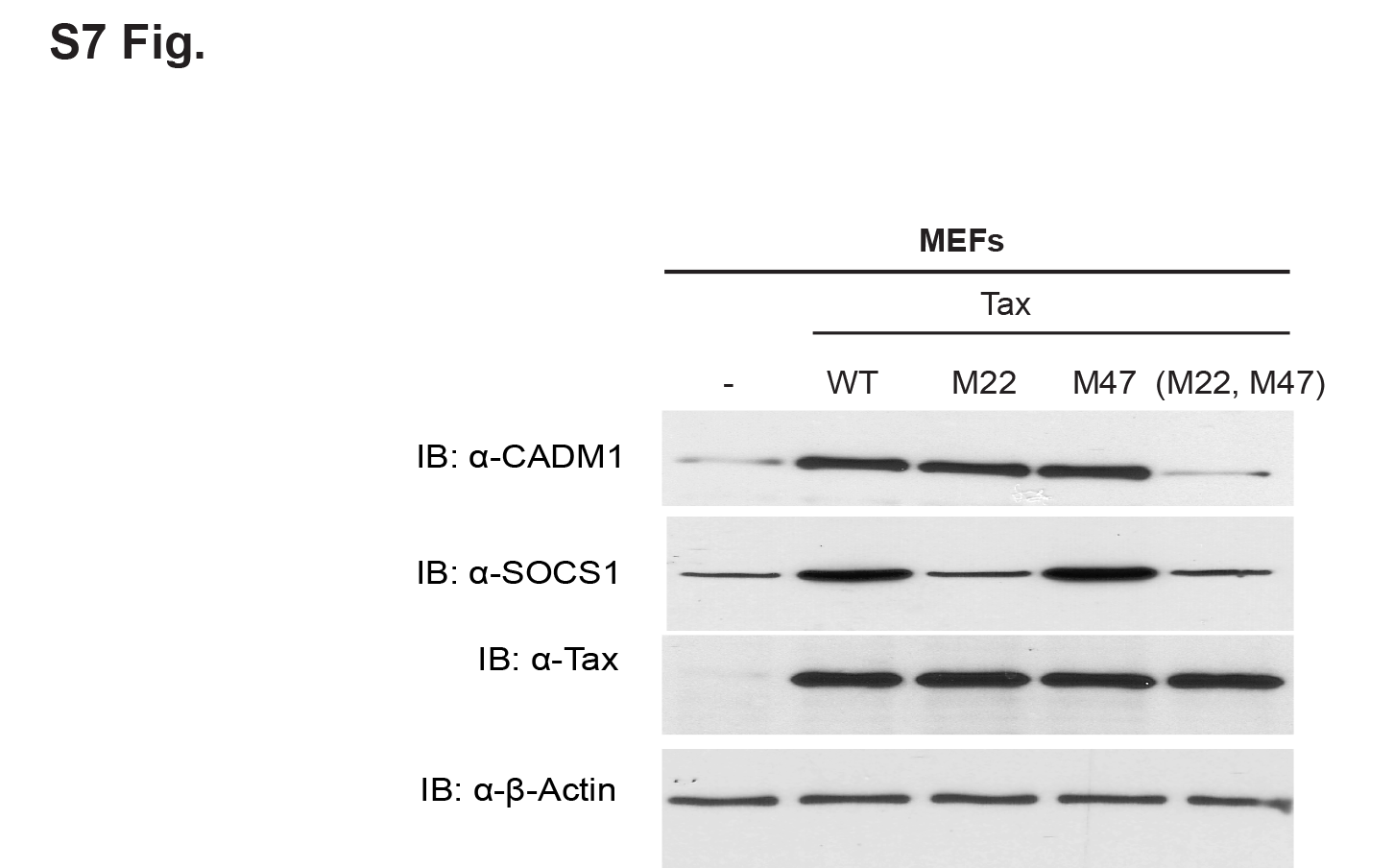

Supplement: S7 Fig — CADM1 expression in lentiviral-transduced empty vector wildtype Tax, Tax single mutants (M22) or (M47), and Tax double mutants (M22 and M47) in primary MEFs was analyzed with anti-CADM1, anti-SOCS1, anti-Tax, and β-actin antibodies. (TIF) [file ppat.1004721.s007.tif]

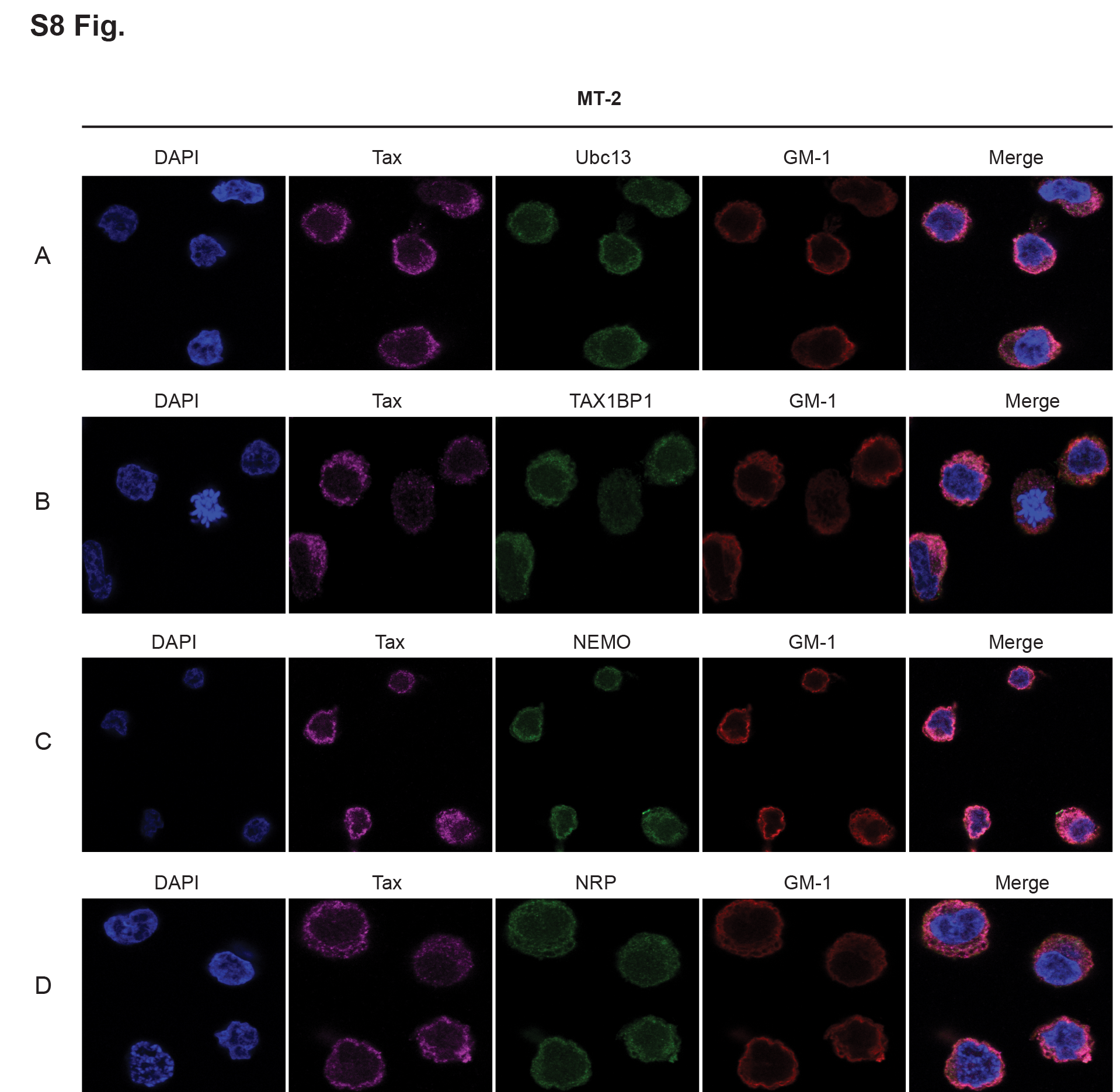

Supplement: S8 Fig — MT-2 cells were co-stained with DAPI, anti-Tax, cholera toxin B conjugated with red fluorescence to detect GM-1, anti-Ubc13 (A), anti-TAX1BP1 (B), anti-NEMO (C), and anti-NRP (D) and subjected to confocal microscopy. (TIF) [file ppat.1004721.s008.tif]

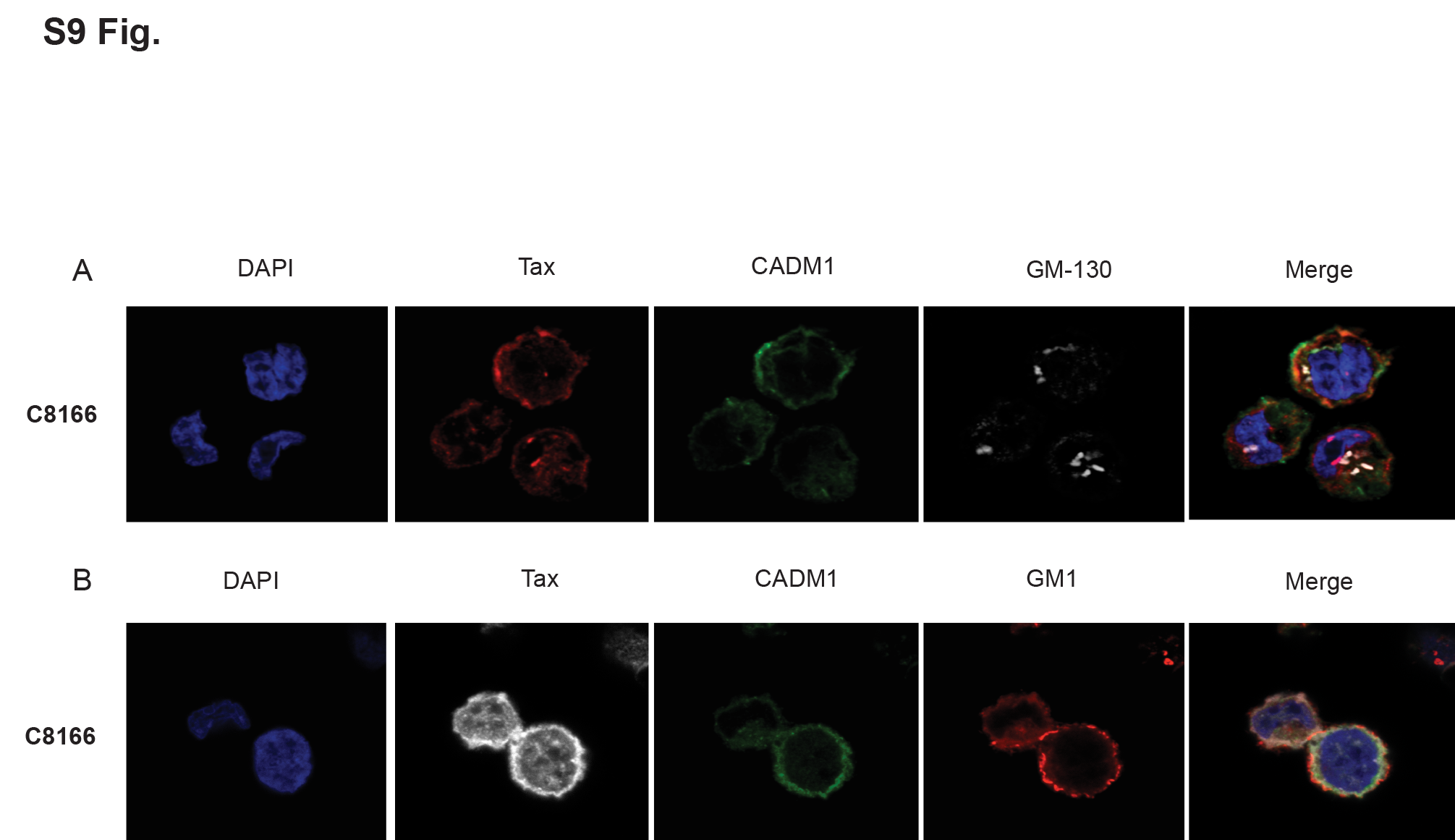

Supplement: S9 Fig — (A) C8166 cells were stained with DAPI, anti-Tax, anti-CADM1, and anti-GM-130, and subjected to confocal microscopy. (B) C8166 cells were stained with DAPI, anti-Tax, anti-CADM1, and cholera toxin B conjugated with red fluorescence to detect GM-1, and subjected to confocal microscopy. (TIF) [file ppat.1004721.s009.tif]

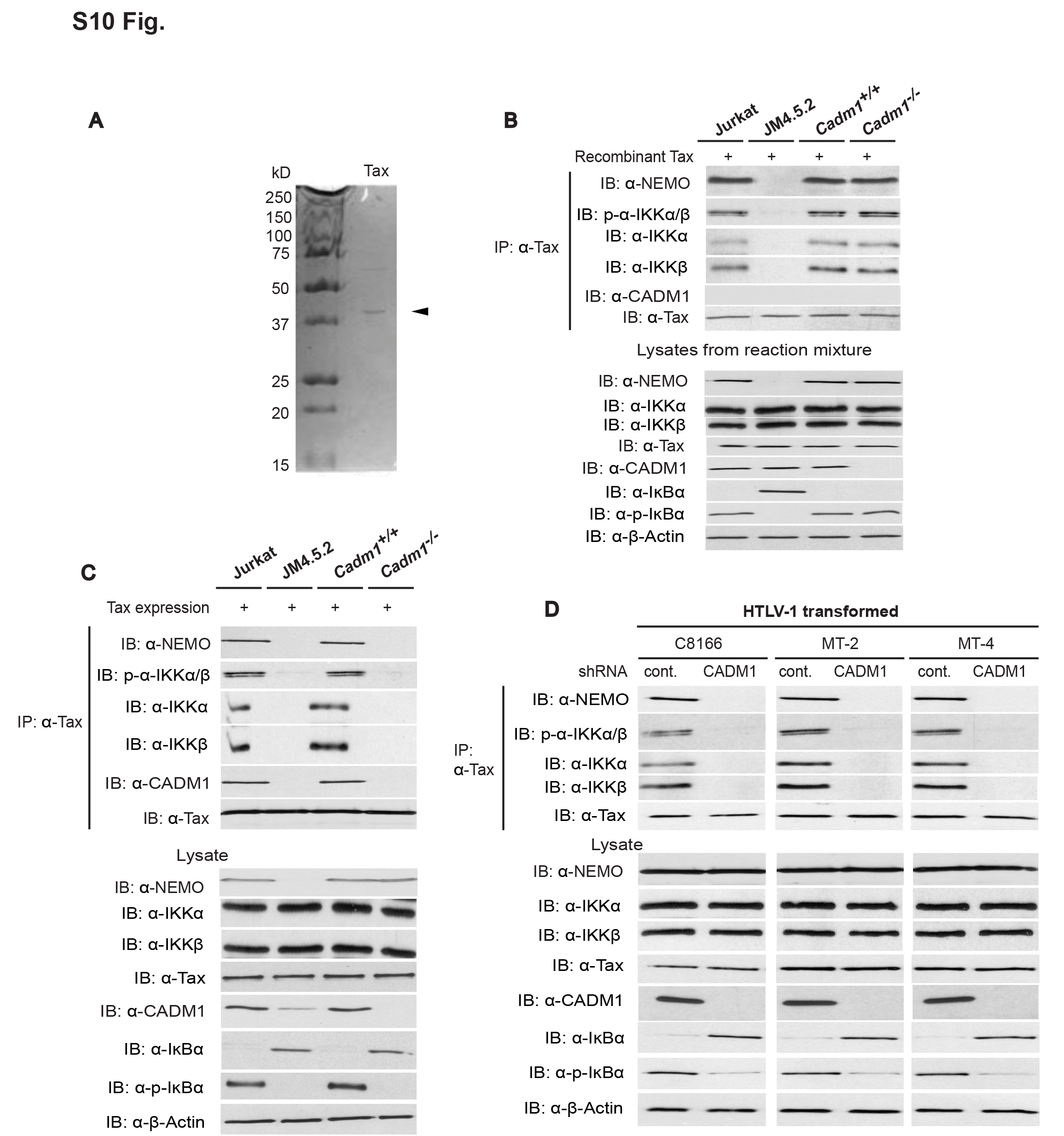

Supplement: S10 Fig — (A) Purified recombinant Tax was analysed by SDS-PAGE and staining with Coomassie brilliant blue. The positions of molecular mass markers (lanes M) (in kilodaltons) are indicated to the left of the gel. An arrow indicates the Tax protein. (B) Induction of IκBα phosphorylation and degradation by recombinant Tax in an in vitro cell free system. Cytosolic extracts (10 mg/ml) from either Jurkat, JM4.5.2, Cadm1 +/+ or Cadm1 −/− cells were incubated with recombinant Tax (250 ng) and ATP (2 nM) at 30°C for 1 hour. Reaction mixture was immunoprecipitated with anti-Tax followed by immunoblotting with anti-NEMO, anti-phospho-IKKα/β, anti-IKKα, anti-IKKβ, anti-CADM1, and anti-Tax. Lysates from these reaction mixtures were further examined for Tax-mediated phosphorylation and degradation of IκBα, expression levels of IKKα, IKKβ, NEMO, CADM1, and β-actin proteins in cytosolic extracts. CADM1 is indispensable for Tax-NEMO binding and Tax-mediated IKK activation, and to induce the first round of IκBα phosphorylation and degradation in intacT-cells. (C) IP-westerns of lysates from lentiviral expressing Tax in either Jurkat, JM4.5.2, Cadm1 +/+ or Cadm1 −/− cells, (D) stably expressing control scrambled shRNA or CADM1 shRNA in Tax expressing HTLV-1 transformed (C1866, MT-2 and MT-4) cell lines, assessed after immunoprecipitation with anti-Tax and immunoblot with anti-NEMO, anti-phospho-IKKα/β, anti-IKKα, anti-IKKβ, anti-CADM1, and anti-Tax. Below (Lysates), immunoblot analysis of total cell lysates with antibodies along left margins with anti-Tax, anti-NEMO, anti-CADM1, anti-p-IκBα anti-IκBα, and anti-β-actin. (TIF) [file ppat.1004721.s010.tif]
